# The therapeutic efficacy of nanoparticles in the treatment of alzheimer’s disease

**DOI:** 10.1007/s13760-025-02905-w

**Published:** 2025-10-13

**Authors:** Thabisa L. Ntondini, Tobeka Naki, Sibusiso Alven

**Affiliations:** 1 Department of Chemistry, University of Fort Hare, Alice Campus, Eastern Cape, Alice, 5700 South Africa; 2 Department of Chemistry, Nelson Mandela University, Gqeberha, 6001 South Africa

**Keywords:** Alzheimer's disease, Blood-brain barriers, Anti-Alzheimer drugs, Nanoparticles dendrimers, And lipid-based nanoparticles

## Abstract

The build-up of beta-amyloid plaques in the brain leads to Alzheimer’s disease (AD), a neurodegenerative condition. AD affects more than 30 million individuals globally every year. No cure for AD has been discovered yet. The available therapeutic options are administered to slow down the progress of the disease. The currently available treatment plans are used to relieve symptoms and improve cognitive abilities, thus slowing progression. Nanotechnology is highly effective and has demonstrated significant benefits across various medical applications. Nanoparticles have been explored as promising drug delivery systems for the targeted delivery of anti-AD therapeutics and for the precise diagnosis of the condition. Nanoparticles, such as dendrimers, lipid-based nanoparticles, polymer-based nanoparticles, and metal-based nanoparticles, have been designed and reported to inhibit Aβ aggregation, fibril formation, and disaggregating mature fibrils, prevent neuroinflammation and Aβ1-42-induced cell damage, treat oxidative stress and lower hallmark of Aβ, and display excellent capability to bypass blood-brain barrier (BBB). This review is focused on the preclinical therapeutic outcomes of nanoparticles and the challenges encountered in the treatment of AD. This review highlights the significant advancements of nanoparticles that are currently undergoing clinical trials for management of AD.

## Introduction

 Alzheimer’s disease (AD) is a brain disorder that slowly weakens memory and thinking skills. It also makes it hard to do even simple tasks [1]. AD is the predominant cause of dementia in older adults. This condition is progressive, meaning symptoms worsen over time. AD significantly impacts various brain functions [2]. AD is classified as mild, moderate, and severe cognitive impairment [3]. Mild AD starts long before any symptoms become visible, such as mild changes in memory and thinking ability, which impacts daily functioning. In moderate cases of AD, the patients become more confused and forgetful, requiring more assistance with daily activities and self-care [4]. In the severe stage of AD, the patient loses the ability to walk, sit, and eat. In some cases, loss of bowel and bladder control is evident, together with the inability to have a conversation, requiring help with all activities.

AD affected over 5.8 million Americans in 2020. AD is uncommon among young people, but common among people older than 65 years old [5]. AD results from an aggregation of amyloid-beta peptides in the brain, namely the neocortical structures and the medial temporal lobe [6, 7]. AD is recognized as a multifactorial condition influenced by various risk factors like advancing age, genetics, and head injuries. While multiple hypotheses have been suggested to explain causes of AD, two primary factors are considered the main contributors: impaired cholinergic function and changes in the manufacturing and operation of amyloid β-protein. However, currently, no theory has been accepted to explain AD pathogenesis. Infections, vascular diseases, and environmental factors (including heavy metals and trace metals) play critical roles in public health and warrant thorough investigation and consideration) [8, 9] have been reported to be contributing factors to AD.

**Fig. 1:**
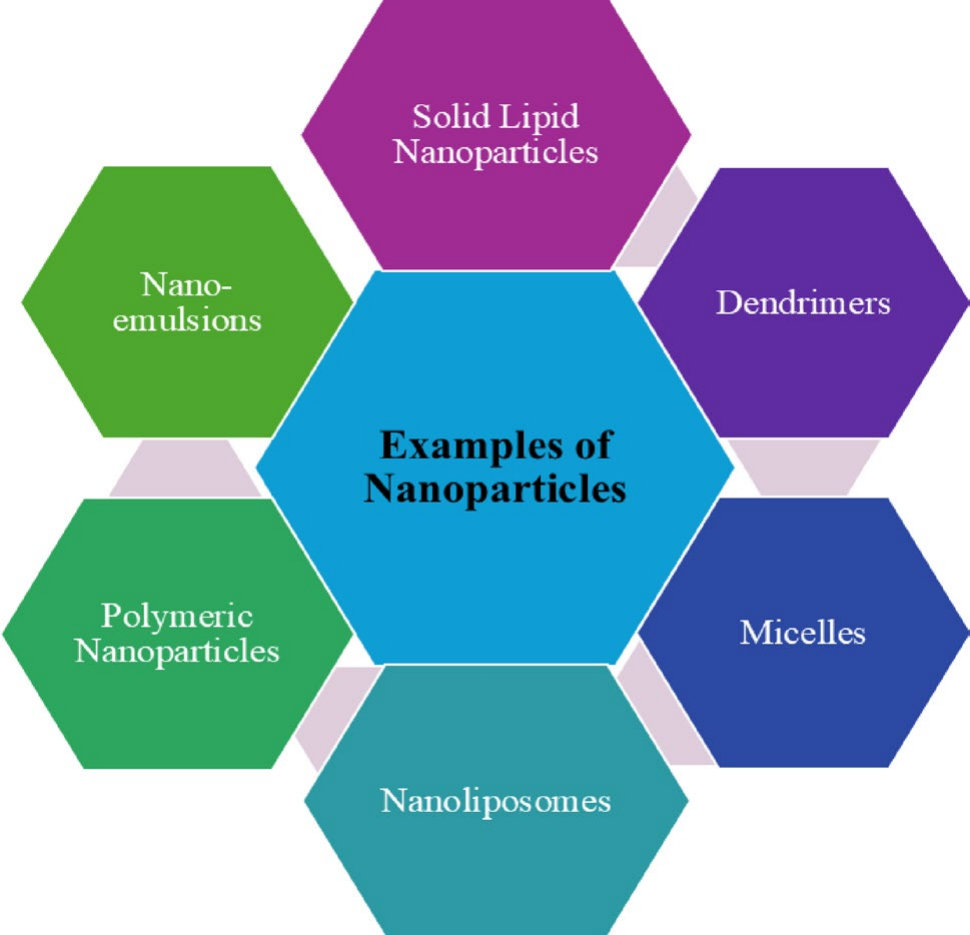
Examples of nanoparticles

There are currently only two classes of drugs that have received approval for treatment of AD: cholinesterase enzyme inhibitors (this includes hybrid analogues, synthetic, and naturally derived) as well as N-methyl D-aspartate (NMDA) antagonists. Certain body processes can harm the cells that produce acetylcholinesterase (AChE). Acetylcholinesterase inhibitors (AChEIs) help by hindering the enzymes AChE and butyrylcholinesterase (BChE) from dissecting acetylcholine (ACh). In AD, the levels of acetylcholine (ACh) increase in the space between nerve cells. However, when N-methyl-D-aspartate receptor (NMDAR) is overactive, it allows too much calcium (Ca2+) to enter the cells. This excess calcium can lead to cell death and problems with how the nerves communicate [10–12]. NMDA antagonists can help by stopping this overactivity, which reduces calcium entry and allows the receptor to work normally again. While these treatments can helping relieve symptoms of AD, they do not cure or prevent the disease [13]. Nanoparticles are extensively employed in biomedical practices, playing a crucial role in diagnosis and treatment of a variety of diseases. Nanoparticles include nanoliposomes, micelles, dendrimers, polymeric nanoparticles, etc. (Fig. 1). Nanoparticles are highly effective in transforming key parameters such as diffusivity, toxicity, solubility, pharmacokinetics, half-life, and diagnostic agents and biodistribution of drugs [14]. Their impact on these factors is significant and well-established. In application of nanoparticles in drug delivery, features like shapes, sizes, materials, drug loading capacity, stability, cell targeting, and release are crucial [15]. Nanoparticles can inhibit the build-up of Aβ (Amyloid-β peptide) when administered to treat AD. They not only stop problems with object-location memory and establishing things, but they can also break apart Aβ fibrils in a helpful way [16]. Nanoparticles have been used for drug delivery to boost the loaded/encapsulated drug’s bioavailability, solubility, stability, and tolerability. Nanomedicine has been designed by researchers to target microbes, with promising results and as a potential strategy to develop antimicrobials, it aims to deliver drugs directly to diseased cell [17]. Determining appropriate analytical tests for the chemical, physical, or biological characterization of nanoparticles presents challenges that require further investigation. This is primarily due to their complexity compared to other pharmaceutical products. Consequently, more sophisticated and comprehensive testing is necessary to ensure thorough and accurate characterization of nanoparticle-based therapeutics [18]. In this review, the advances in the design of nanoparticles and their efficacy in the management of AD will be discussed along with their limitations. This review outlines the effects of AD; how it can be managed via available therapeutic options and the modifications of these available therapeutic options.

## Anti-Alzheimer drugs and their mechanisms of action

**Fig. 2:**
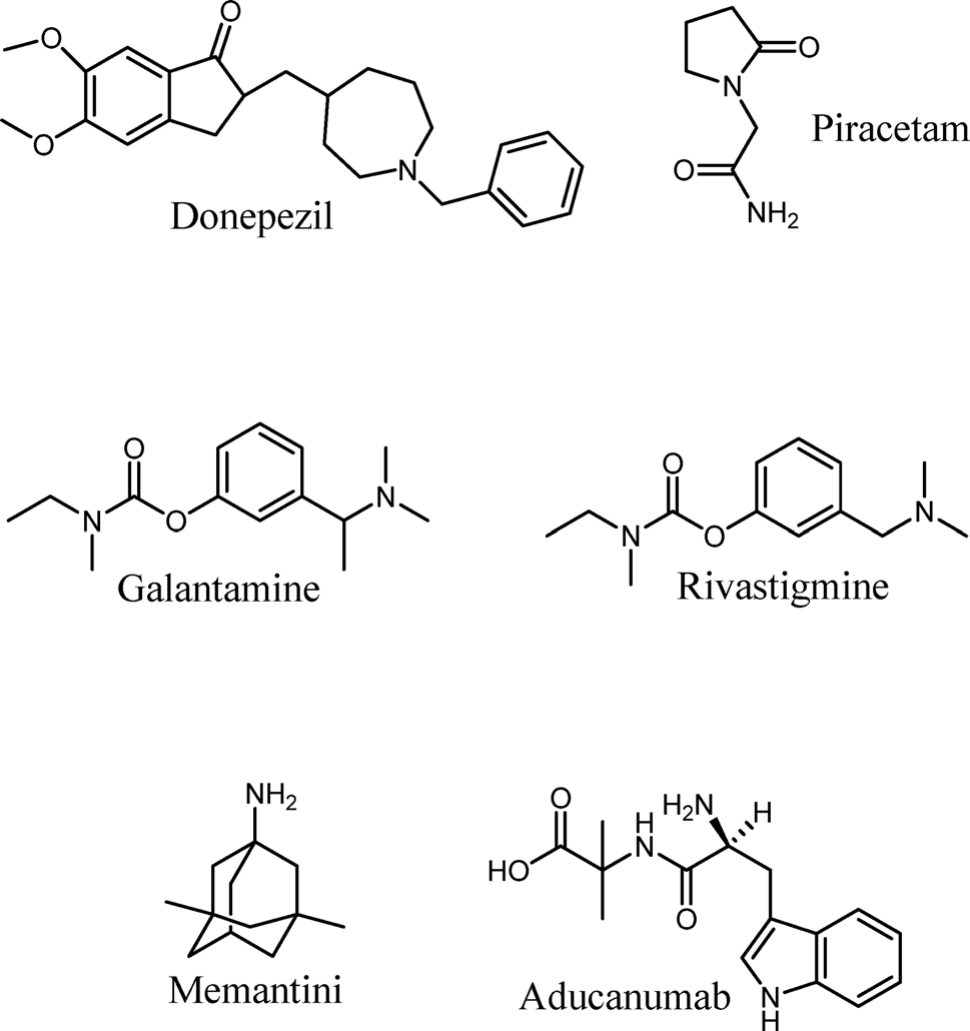
Molecular structures of anti-Alzheimer drugs

According to Pardo-Moreno et al., the U.S Food and Drug Administration (FDA) has approved several medications for AD treatment, recognized for their efficacy and safety in managing symptoms. These include donepezil, rivastigmine and galantamine (cholinesterase inhibitors), memantine (an NMDA receptor antagonist), aducanumab and lecanemab (anti-amyloid monoclonal antibodies) (Fig. 2) [19, 20]. Acetylcholinesterase inhibitors (AChEIs) used for the treatment of AD are rivastigmine, donepezil, and galantamine. AChEIs inhibit ACh breakdown in the brain of AD patients and inhibiting the enzyme acetylcholinesterase in the synaptic cleft [21]. They also improve the central cholinergic neurotransmission, reducing a decline in cognition functions. AChEIs therapeutics are administered immediately after the diagnosis. AChEIs such as rivastigmine and Donepezil are administered to manage mild, moderate critical forms of AD. Galantamine is mostly administered to treat mild and moderate AD [21]. Non-competitive low-affinity N-methyl-D-aspartate-receptor open-channel blockers such as memantine affect glutamatergic transmission and are effective for treating moderate to severe AD [22].

Memantine is a clinically effective therapeutic administered for a variety of neurological disorders, including AD [23]. However, memantine combination therapy and monotherapy are associated with adverse events like drowsiness. It is well-tolerated, and its safety has been compared to that of a placebo [23]. Memantine is a proven and effective uncompetitive antagonist of N-methyl-D-aspartate receptor, known for its moderate affinity [24, 25]. Memantine significantly enhances cognitive function and effectively addresses behavioral disturbances, outperforming placebo both on its own and in combination with donepezil [26]. Memantine confidently functions as an uncompetitive antagonist of NMDA receptors, which are a subtype of glutamate receptors in the central nervous system (CNS) [24]. It counteracts excessive stimulation [24]. It further induces antagonist activity at nicotinic acetylcholine receptors and serotonergic type 3 (5-HT3) [24].

Donepezil is sold under the brand name Adlarity, featuring a reliable transdermal delivery system that is administered weekly on the back, buttocks or thighs [20]. Donepezil is accessible in 10-mg/d plus 5-mg/d dosages, restoring oral donepezil at the same dosages [20]. According to Larkin et al., this system was developed to continuously deliver medication via the skin plus keeping constant levels needed for efficacious treatment [20]. This type of delivery can lower the risk of adverse gastrointestinal effects that result from oral donepezil. Donepezil improves patients cognitive functions [26]. Its administration in high doses is mostly prescribed for patients whose therapeutic response is poor at lower doses and who are at risk of increasing cholinergic side effects that include nausea, dizziness, anorexia, and vomiting [27].

Donepezil binds and reversibly inhibits enzymes known as cholinesterases, particularly acetylcholinesterase. It prevents acetylcholine hydrolysis, which boosts acetylcholine levels at cholinergic synapses [28]. Galantamine is a type of acetylcholinesterase inhibitor. Galantamine is well-known for its effectiveness in slowing cognitive decline [29]. This medication is used to treat early-stage AD and memory problems. However, it is less effective for advanced stages of dementia. It works by increasing a neurotransmitter called acetylcholine. Additionally, it stops an enzyme called acetylcholinesterase from breaking down acetylcholine. It was first marketed as *Nivalin* ^®^ in Bulgaria and other Eastern European countries. Some of its adverse effects are muscle weakness, muscular dystrophy, trigeminal neuralgia, and peripheral nerve inflammation [30].

Galantamine is a unique medication that acts as a powerful allosteric potentiator of the α4β2 and presynaptic α7 nicotinic acetylcholine receptors. Its mechanism significantly enhances release of acetylcholine from presynaptic neurons, underscoring the clinical importance of its dual mode of action [31]. Aducanumab is a fully human IgG1 monoclonal antibody that targets a conformational epitope on β-amyloid, a protein associated with AD. This targeted approach is integral to advancing our understanding and treatment of this complex neurodegenerative condition [32]. It was accepted for the treatment of AD in June 2021 by the Food and Drug Administration [33]. Aducanumab effectively targets and binds to amyloid aggregates in both their oligomeric and fibrillar forms, demonstrating a distinct selectivity over amyloid monomers [34]. Unique amino acid interactions facilitate shallow and compact binding, significantly reducing interactions due to the limited availability of epitopes on the monomers. In contrast, the strong affinity for aggregates arises from a higher concentration of specific epitopes for the monoclonal antibody, leading to an even greater binding affinity [32, 35].

Rivastigmine (RHT) is an effective cholinesterase inhibitor utilized in treatment of mild to moderate AD through oral administration. Its efficacy and clinical application make it a valuable option in managing this condition [36]. Oral dosing, while commonly used, tends to have a slower absorption rate and can lead to significant systemic side effects [36]. Compared with a placebo, better outcomes were noted for the rate of decline of cognitive functioning and activities of daily living, even though outcomes were small with uncertain clinical significance [37]. Additionally, rivastigmine clearly provided benefits in the clinician’s global assessment outcomes [37]. Rivastigmine effectively inhibits both acetylcholinesterase and butyrylcholinesterase by forming a covalent bond with the active sites of these enzymes, thereby blocking their function [38]. It stunts hydrolytic activity of AChE and BChE while binding to catalytic sites. This medication is effective in treating dementia associated with Alzheimer’s and Parkinson’s diseases [39].

## Blood–brain barrier and delivery of drugs to the brain

The blood-brain barrier (BBB) is a restrictive microvasculature lining of central nervous system (CNS) that modulates the uptake of cells, ions, and molecules between blood and CNS [40, 41]. It is made up of a monolayer of endothelial cells, which are reinforced by pericytes, microglial cells, and astrocyte end-feet [40]. The endothelial cells are joined by tight junctions, providing a paracellular railing that merges endothelial cells into the abluminal membrane compartments and the distant luminal. The tight junctions are composed of a network of transmembrane proteins. The paracellular barrier enhances endothelial delivery by regulating molecule movement between the blood and the brain [40, 41].

BBB is also crucial in controlling CNS homeostasis, which is essential to neuronal function and protection of the CNS from pathogens, disease, inflammation, toxins, as well as injury [42]. This protective feature of the BBB also acts as a hindrance to effective drug delivery to the CNS. Several researchers have explored systems that bypass or control the BBB to deliver therapeutics [43]. Neurological conditions such as AD result in the dysfunction of BBB [44]. BBB functions as crucial interface between CNS and peripheral tissues. Changes in BBB are integral to onset and progression of AD, establishing it as prominent therapeutic target. Moreover, the BBB poses a substantial dispute to drug delivery for brain tissue, making it a key consideration in the treatment of AD. Several approaches have been explored to target BBB [45]. Nanoparticles are nanosized carriers designed for targeted cellular uptake, making them potential systems for drug delivery via the BBB [46].

- **Transcellular routes**.

Passive diffusion serves as the primary transcellular route, but it is only effective for therapeutics that possess the requisite physicochemical characteristics, including appropriate size and shape, hydrogen bonding potential, and hydrophobicity. CNS therapeutic molecules must navigate two lipid bilayer membranes, the apical/luminal membrane and the basolateral/abluminal membrane, both of which present significant rate-limiting obstacles to reaching their target site [47]. The apical membrane contains a variety of proteins, including receptors, enzymes, and transporters. Additionally, the passive diffusion of drugs across the membrane is primarily restricted by the dense arrangement of the phospholipid’s polar head groups, followed sequentially by the nonpolar tails of the phospholipids, which form a hydrophobic barrier [48].

- **Adsorptive-mediated transcytosis (AMT)**.

The concept of delivering antidepressant medications via BBB has been informed by the insightful observation that polycationic proteins, such as protamine, possess the unique ability to bind to endothelial cell surface and facilitate penetration of BBB. This understanding opens important avenues for further exploration and development in the field [49]. AMT does not require specific receptors on the plasma membrane. Instead, endocytosis occurs when polycationic substances interact with the negative charges present on the endothelial surface [50].

- **Receptor-mediated transport (RMT)**.

Specialized receptors that facilitate the transport of cargo from the luminal to the abluminal sides of BBB have paved the way for innovative therapeutic delivery strategies to this highly restricted region. The specific pathways that these receptors participate in are referred to as receptor-mediated transcytosis (RMT), these receptors are commonly known as “RMT receptors [51].

RMT is a complex transcellular activity that takes place in polarized cells, allowing naturally transpiring macromolecules to navigate around physiological barriers and access constricted areas of the body. Notable illustrations of these macromolecules include low-density lipoproteins (LDLs), transferrin (Tf), insulin (INS), and insulin-like growth factors 1 and 2 (IGF1, IGF2). Consequently, RMT receptors present attractive targets for the development of targeted antibodies and peptides that can act as macromolecular Trojan horses, facilitating delivery of therapeutics across BBB [52].

- **Cell-mediated endocytosis**.

Receptor-mediated endocytosis is a highly effective process in which macromolecules bind to specific transmembrane receptor proteins. These macromolecules assemble in coated pits and seamlessly invade cell as receptor-macromolecule complexes within clathrin-coated vesicles. This mechanism is essential for the selective uptake of important substances by the cell. Endocytosis is the process by which cells consume molecules, fluid, and particles. Endocytosis is the process where specific regions of plasma membrane invaginate and successfully pinch off to create endocytic vesicles. Numerous molecules and particles that are taken up by this process ultimately reach the lysosomes, where they are devalued. Endocytosis is a process that can occur both spontaneously and in response to external signals. This phenomenon is so widespread among various cell types that a substantial portion of the plasma membrane is internalized on an hourly basis [53].

- **Use of peptide vectors**.

Research demonstrates that small peptides effectively cross biological membranes through this mechanism, making them excellent candidates for drug delivery. A prime example of this is the use of diketopiperazines (DKPs), which have shown significant potential in this area [54]. Highly stable cyclic dipeptides have been shown to enhance the passive diffusion transport of two small compounds of therapeutic interest, L-dopa and baicalin, as demonstrated by the PAMPA assay, which is recognized as the gold standard for evaluating this mechanism. Additionally, this group of compounds was utilized to deliver hexapeptide that can stunt Tau accumulation in vivo in mouse models [55].

- **Transnasal drug delivery to the brain**.

Drugs effectively bypass BBB through the nasal mucosa, allowing for direct delivery to nerves in the brain and spinal cord via neural pathways [56]. Once delivered to brain, drugs effectively disperse using intracellular or extracellular delivery mechanisms. Intracellular delivery allows drugs to enter and exit neurons through processes like cytokinesis and receptor-mediated transport, predominantly via axons that connect to the central nervous system (CNS). This efficient routing ensures that therapeutic agents reach their intended targets within brain. Fluid movement improves the dispersion of the medicine. Extracellular delivery takes place when the medicine penetrates nasal epithelium, entering lamina propria, where neurons are situated. From there, drug is carried along neuronal axons via overall flow transport process through perineural channels, facilitating its transfer from one neuron to another [56].

## Nanoparticles for drug delivery to manage AD

### Solid lipid nanoparticles (SLN’s)

**Fig. 3:**
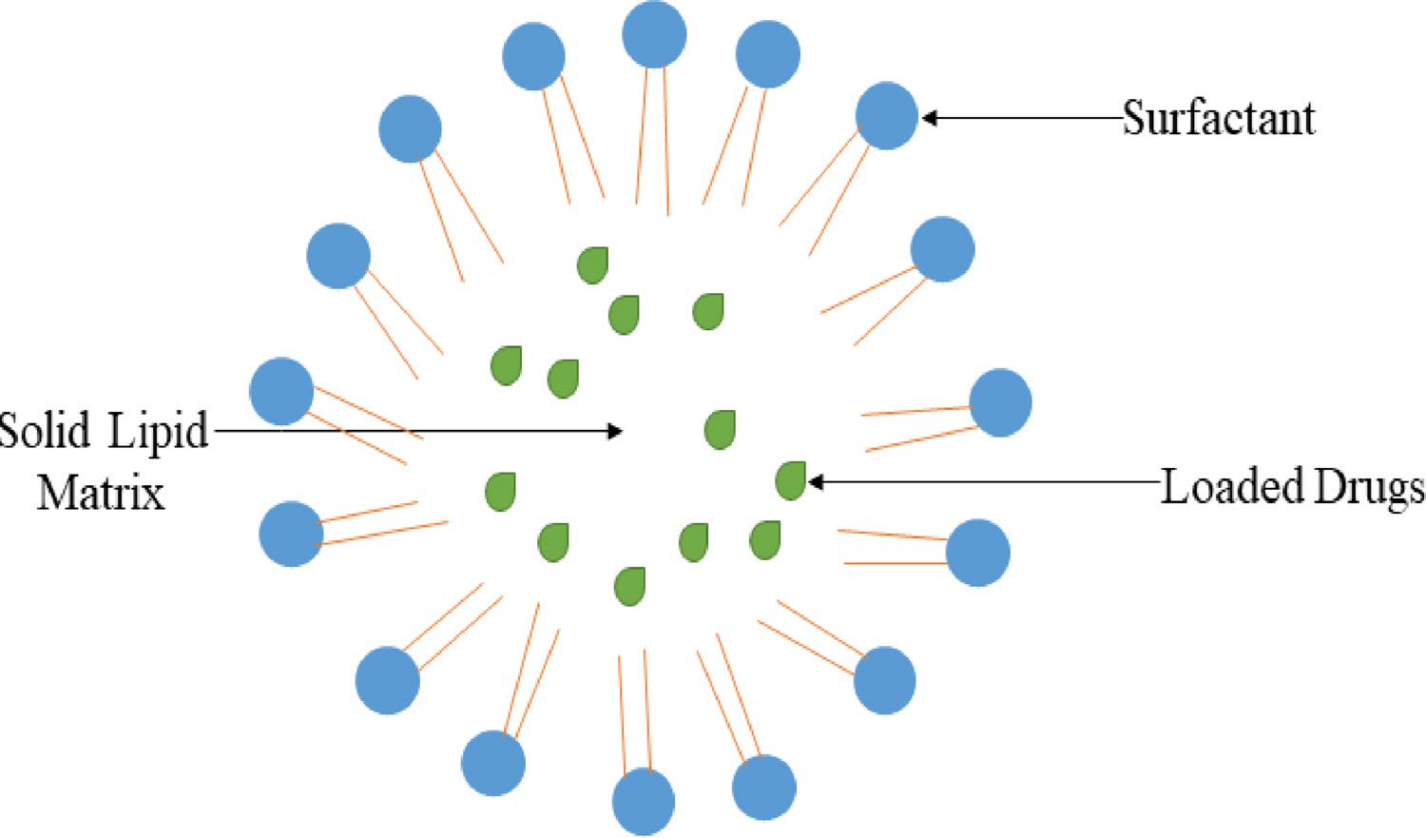
Structure of SLNs

SLN’s are undoubtedly one of the safest and most cost-effective options for drug delivery. Providing a non-toxic, safe, and efficient means of treating neurological disorders by penetrating BBB. The functionality and effectiveness of SLN’s are influenced by their composition, size, structure, physicochemical properties, and the manufacturing methods employed in their production. It is essential to highlight the advanced manufacturing technologies utilized in the development of SLN’s within the drug delivery sector. The latest lipid nanoparticles have progressively addressed and surpassed the limitations of earlier SLN formulations [57]. SLN’s have several limitations, like low drug loading efficiency and risk of drug expulsion due to crystallization during storage. Additionally, lipid dispersions contain a high-water content and offer restricted transdermal drug delivery. SLN’s are spherical nanocarriers composed of a solid lipid core matrix (Fig. 3) [58]. Table 1provides a summarized overview of the research on nanoparticles utilized for drug delivery inthe treatment of AD.

They are likely preferred for drug delivery in penetrating the BBB. They also have ability to solubilize lipophilic molecules [59]. They consist of a blend of solid lipids stabilized by surfactants within an aqueous medium, with diameters ranging from 40 to 1000 nm. By replacing 30% of lipid mass with liquid lipids, a different type of SLN’s can be produced; these are referred to as nanostructured lipid carriers. SLN’s have numerous advantages, including biocompatibility, less toxicity, drug oxidation defense, chemical resistance, high drug loading, and numerous. As a result, they can be used in cosmetics and pharmaceutical formulations [60].

Naser et al. reported that sterylamine-based SLN’s containing antipsychotic drug, clozapine. They are specifically engineered to deliver drugs effectively to the brain via intravenous and intraduodenal routes. Quercetin-loaded SLN’s play a crucial role in effectively managing AD [61]. Campisi and colleagues developed a systemic administration of SLN’s to facilitate the delivery of curcumin and its effects on TG2 isoform expression levels in wild-type (WT) and TgCRND8 (Tg) mice. The study concluded by suggesting that SLN’s-CUR could be an innovative tool for treating AD [62]. In another study, Albino Wistar rats were confidently utilized for comprehensive biodistribution and pharmacokinetic studies of intranasal dupilumab-SLN’s. The research findings showed that the most effective dupilumab-SLN had a shelf life of 2.29 years. The research revealed SLN’s are effective in delivering dupilumab (DPL) to brain [63]. Yasir et al. developed SLN’s to evaluate their effectiveness in delivering donepezil directly to brain via nasal route. In both in vivo and in vitro release tests, donepezil SLNs demonstrated a superior release rate compared to intravenous (IV) and intranasal (IN) donepezil solutions. This improved release rate may be due to the SLN’s capacity to shield the encapsulated drug from degradation and enhance transport by P-glycoprotein (P-gp) efflux proteins [64].

Topal et al.. conducted a study utilizing Albino Wistar rats to investigate pharmacokinetics and biodistribution of intranasal DPL-SLN’s. This study showed area under the curve (AUC) to be 2.61 times higher than intravenously (i.v.) administered DPL-Sol, plus 2.26 times higher than intranasally administered DPL-Sol. Moreover, scintigraphy investigation of rabbit’s brain displayed drug’s location [65]. In conclusion, optimal DPL-SLN had a shelf-life of 2.29 years, revealing that SLN’s are useful in delivering DPL to brain. Dara et al. showed an encapsulation of Erythropoietin (EPO) to SLN’s can lower Aβ deposition, oxidative stress, and increase spatial memory [66]. Chauhan and Sharma formulated transdermal patches of rivastigmine-loaded nanolipid carriers to treat dementia [67]. According to Mendes et al., an in vitro evaluation of donepezil-loaded lipid gels exhibited improved transdermal delivery of donepezil for treating AD [68].

**Fig. 4:**
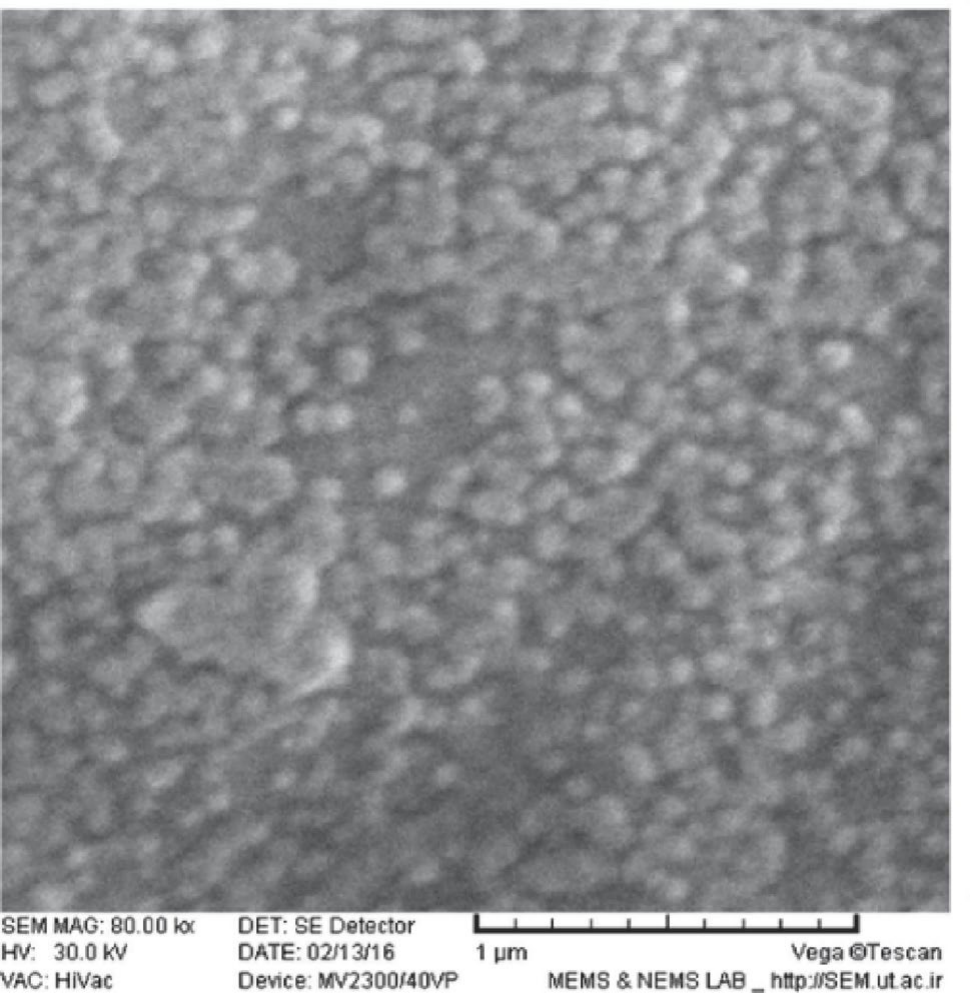
SEM image of EPO-SLN. Reproduced with copyright permission from Elsevier [66]

A study conducted by Dara et al.. revealed that application of superparamagnetic liposomes loaded with hematopoietic factor erythropoietin (SLN-EPO) effectively enhanced memory deficits in a rat model induced by AD. The SEM micrograph of SNL’s showed spherically shaped nanoparticles with an average diameter of 219.9 ± 15.6 nm (Fig. 4) while DLS analysis displayed a narrow PDI of 0.187 ± 0.03 and surface charge − 22.4 ± 0.8 mV, revealing their capacity to pass the BBB and deliver bioactive agents to the brain [66]. Furthermore, EPO-loaded SLN’s have ability to improve BBB penetration, antioxidant properties, inhibit Aβ plaque deposition, as well as lower ADP/ATP ratio. According to a report by Pinheiro et al.. a formulation of Transferrin-functionalized SLN’s of quercetin was used to improve earmarking capability and antioxidant activity, which relieved AD symptoms [69]. Poudel et al. reported an in vivo plus in vitro test of lactoferrin-modified SLN’s loaded with curcumin, demonstrating proof of increased targetability as well as activity in the brain for treating AD [70]. Hu et al. designed SLN’s loaded with ferulic acid. The SLN’s displayed enhanced penetration ability and improved memory functions with excellent nasal mucosal adhesion [71]. Several studies on SLN reveal their capability to deliver drugs, improve memory efficiency, and enhance BBB penetration and targeting capability.

### Nanostructured lipid carriers (NLC’s)

**Fig. 5:**
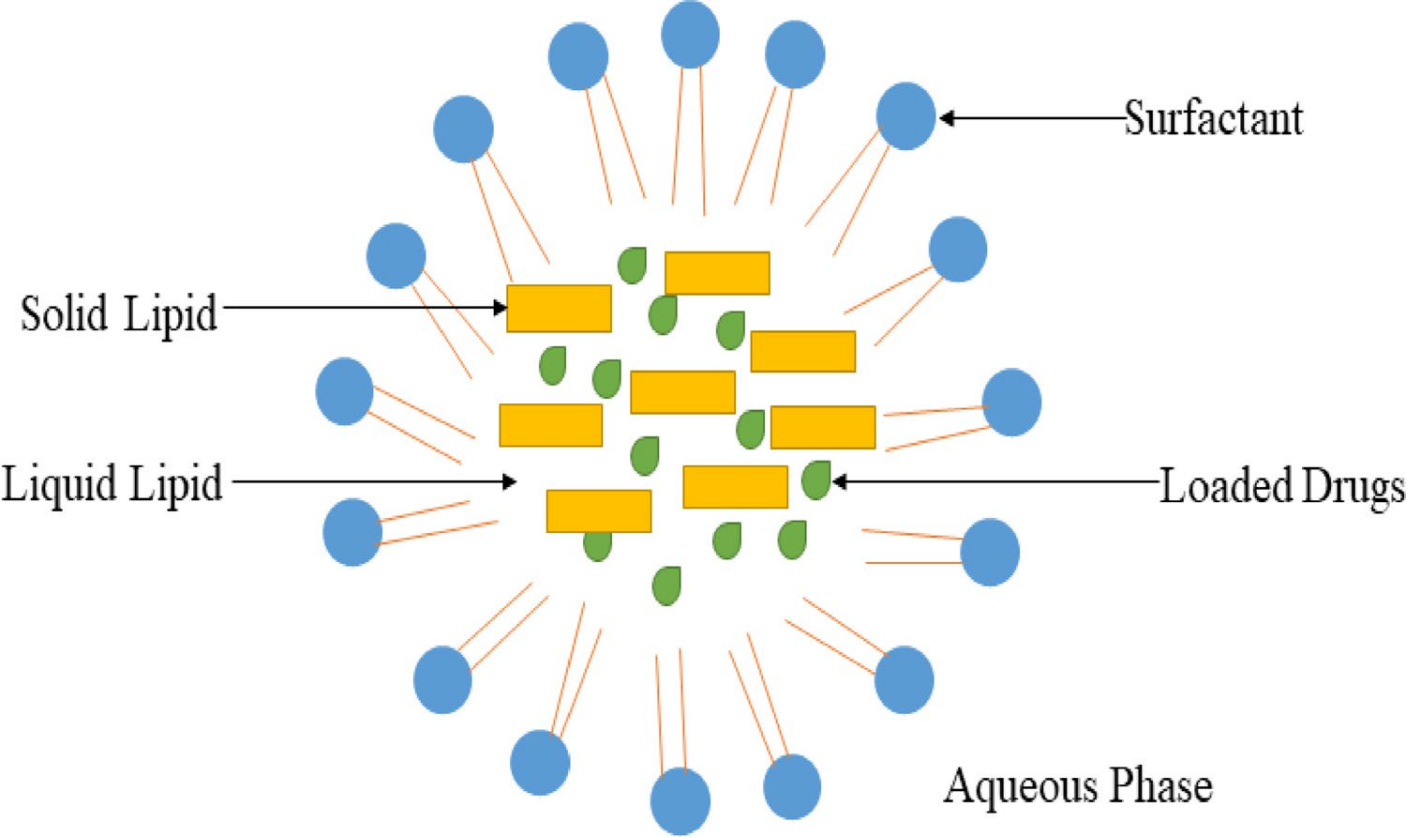
Structure of Nanostructured lipid carriers

NLC’s arise as optimistic targeted medicine delivery systems and consist of an inner lipid matrix of solid plus liquid lipids (Fig. 5) [63]. They have a promising loading capacity and are stable [72]. They are applied for delivering medicine coming out of the nose to brain and explored for treating AD linked with microglial activation [73]. NLC’s offer several advantages, including easy producing, less toxicity, and physical resistance. They allow for custom-tailored drug release and have high drug entrapment capabilities. Additionally, NLC’s prevent drug leaching during storage and improve both drug solubility and stability. These magnificent characteristic provide a significant advantage over other drug delivery systems [74]. Their lipophilic nature and small particle/droplet size help them target the brain effectively [75]. These characteristics allow the drug to penetrate BBB, and its encapsulation in the lipid matrix prevents it from enzymatic degradation. The protection enables the drug to reach the brain at therapeutic levels [76].

**Fig. 6:**
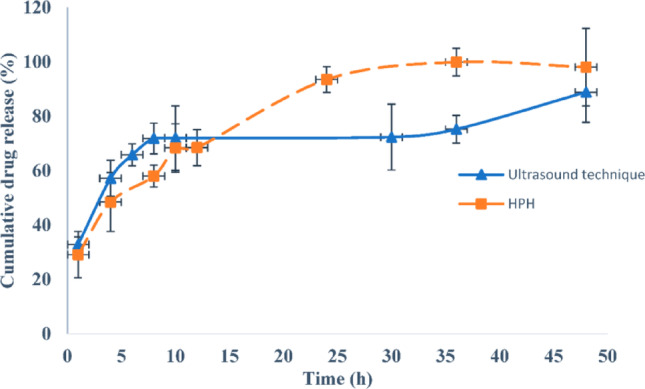
The cumulative drug release percentages for rivastigmine-loaded NLC’s in a simulated nasal electrolyte solution (pH 6.4) were rigorously assessed, showcasing the effectiveness of both ultrasound and high-pressure homogenization (HPH) production techniques. This data is reproduced with copyright permission from MDPI [77]

Cunha et al. executed a dual optimization of a formulation using rivastigmine-loaded NLC’s for immediate medicine delivery from intranasal. They employed Quality by Design (QbD) method, with quality intent product profile (QTPP) established as a key requirement for effective nose-to-brain delivery [77]. The in vitro drug declare profile exhibited beginning expeditions let out of rivastigmine from CS-NLC, which was associated with the diffusion of the drug from the surface of nanocarriers, followed by a prolonged drug release (Fig. 6). These results revealed that these CS-NLC’s can offer rapid therapeutic efficacy that can be beneficial for AD by persuading fast drug release and continuously sustaining therapeutic activity after. The optimized formulation of rivastigmine-loaded NLC’s, developed through the high-pressure homogenization (HPH) method, exhibits remarkable stability and represents a promising alternative for the efficient delivery of rivastigmine directly to the brain via the nasal route. The lipophilic nature and small particle/droplet magnitude of it make it effective for targeting the brain [77]. Wavikar et al. mentioned that a new in situ gelling system was created. In this system, RV-NLCs containing 6.25% w/w resveratrol (RV) were developed with other modifications to the formulation process. The outcomes indicated a sustained release of IN and IV NLC’s when compared to RV solutions administered by same routes. Additionally, the NLC’s exhibited notably higher AUC and T1/2 [78].

Sood et al. successfully developed NLC’s loaded with curcumin and donepezil specifically for brain delivery via nasal path. The NLC’s demonstrated a particle magnitude of under 50 nm, which positions them as highly effective for nasal administration. In vivo pharmacokinetic studies clearly exhibited that this procedure achieved a notable result; the drug demonstrates a notably higher concentration in the brain when delivered via this method in comparison to intravenous route, along with a sluggish clearance rate [79]. Gartziandia et al. said a good formulation of chitosan-coated NLC’s has been developed to assess brain delivery effectiveness after intranasal administration. The study on the distribution of CS-NLC’s loaded with near-infrared dye clearly demonstrates that these particles are effectively delivered to the brain following intranasal administration. This efficacy is attributed to chitosan’s strong mucoadhesive properties, which significantly reduce mucociliary clearance and enable medicine to reach olfactory region with great success [80].

According to Malvajerd et al.. Curcumin-loaded NLCs can treat oxidative stress conditions in AD [81]. Curcumin-loaded NLC’s increased curcumin bioavailability in the brain as well as lowered hallmark of Aβ in AD [81]. Ullah et al.. reported loading of Lipid nano-capsules with a lipid core and with indomethacin, having been evaluated for their ability to inhibit neuroinflammation and Aβ1–42-induced cell damage [82]. Results showed that Ind-LNC’s can prevent neuroinflammation induced by Aβ1–42 in organotypic hippocampal cultures, as well as decrease A-induced cell death. NLS nanoparticles have ability to treat oxidative stress, lower hallmark of Aβ, prevent neuroinflammation also decrease A-induced cell death [82].

### Microemulsion/Nano-emulsion (NE)

**Fig. 7:**
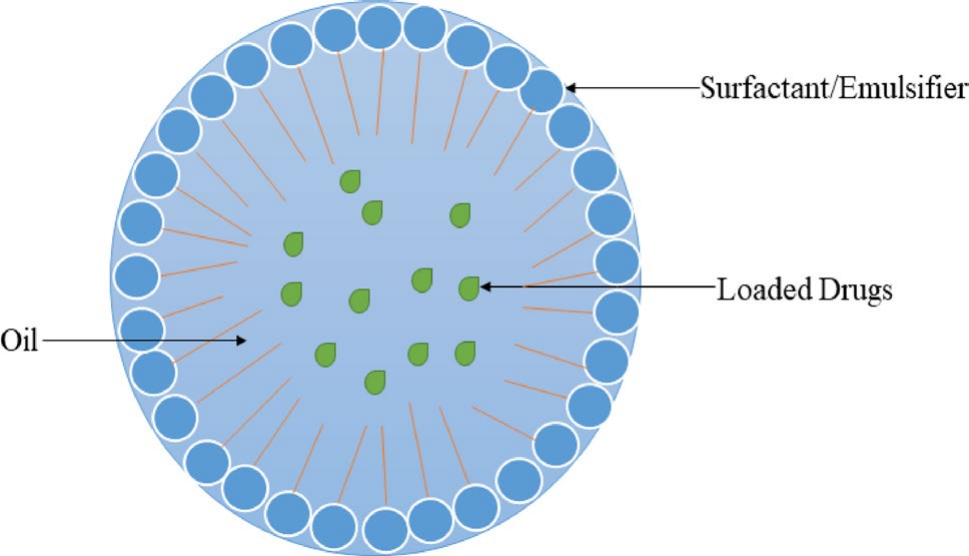
Structure of NE

NE is an emulsion-based nanocarrier system that is applied in delivering drugs in AD (Fig. 7) [83] and earmarked drug delivery in AD, plus increasing the effectiveness of anti-AD drugs [83]. They are widely used as medicine transporter systems for contemporary, oral, and parenteral drug administration, providing numerous benefits like a ease of composition, spontaneous formation, scaling-up thermodynamic stability, improved medicine dissolution, plus bioavailability [84]. NE significantly improve drug therapeutic efficacy while also reducing volume of drug delivery vehicle, thereby reducing toxicity [84]. Patil and colleagues noted that a cubosomal-loaded mucoadhesive formulation of donepezil was created and evaluated for targeted delivery in treating of AD [85]. The outcomes of the in vivo including in vitro tests exhibited optimistic outcomes of the emulsions in AD pathologies. Kell et al.. examined the potency of lactoferrin-modified NE in an in vitro brain model [86]. The findings illustrated the promising targeted delivery of the drug to the brain and enhanced medicine release into the brain tissue. Mir et al. confirmed that utilizing nanogels composed of tripolyphosphate and chitosan to deliver deferoxamine is an impactful treatment for AD [63].

Kaura et al.. studies prepared NE loaded with memantine using homogenization and ultrasonication [87]. This formulation was able to cross BBB when administered through the intranasal route and increased anti-AD impact than the conventional dosage form [87]. Kaura et al.. prepared NE comprising DPL hydrochloride using glycerol and labrasol at 10% w/w. The NE formulation displayed radical scavenging and antioxidant effects [88]. DeRidder et al.. loaded NE with memantine for a rapid medicine let out rate of 80% within 6 h and 77% drug release at 6 h for memantine packed into dendrimers [88]. Moreover, loading NE with donepezil shows a higher zeta potential (− 10.7 mV) than SLN, PNP, and liposomes [89]. Jiang et al.. report a loading of huperazine (Hup A) with lactoferrin (Lf) and -conjugated to NE [90]. The findings displayed that Hup A-loaded NE can amplify the penetration in BBB via intranasal administration as well as improve safety, targeting ability, and action duration [89]. NE has been reported by several researchers to display a high capability to cross BBB when administered intranasally, releasing drugs at a rapid rate.

### Dendrimers

**Fig. 8:**
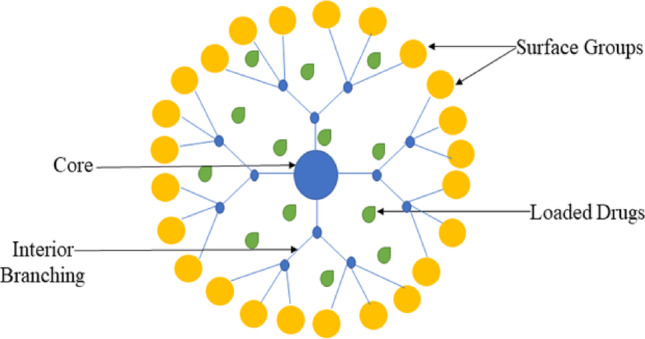
Structure of Dendrimers

Dendrimers arise as nanometer-sized synthetic polymeric macromolecules of multitudinous mostly branched monomers that emerge radially from the mid-core (Fig. 8). Their structure provides several benefits, including controllable magnitude and monodisperse, modifiable exterior, functionality, water solubilization, multivalency, and accessible interior cavity for medicine delivery. The resulting spherical macromolecular structure is comparable in magnitude to albumin and hemoglobin, but compact than multimers such as the IgM antibody complex [91]. Dendrimer architecture and flexibility have enabled greater progress in their application for intended drug delivery. Current research is focused on the application of biocompatible dendrimers for the targeted delivery of chemotherapeutic agents in cancer therapy, specifically cisplatin and doxorubicin [92]. Dendrimers are medicine delivery systems that meet supreme standards, including being biocompatible, non-toxic, not eliciting an immune response, naturally degradable, and avoiding recognition by immune mechanisms in the body [93]. Dendrimers have the potential to be good drug carriers because of their precise structures and a variety of different types of groups [94].

Igartua and colleagues created dendrimers with a maltose histidine shell (G4HisMal) and poly (propylene imine) core, which might exhibit significant relief of AD symptoms like memory dysfunction. Moreover, because of their enhanced bioavailability, solubility, and capability to cross BBB, they can attack blemished parts of the brain [95]. According to the findings presented by Gothwal et al., the administration of dendrimers comprising polyamidoamine (PAMAM) conjugated with lactoferrin (Lf), which were loaded with memantine (MEM-PAMAM-Lf), resulted in significant improvements in cognition, memory, and behavioral patterns in rats induced with AD through aluminum chloride [96]. DeRidder announced that D-tesaglitazar, a novel dendrimer-PPARα/γ binary agonist, induces a shift from ‘M1 to M2’ phenotype, along with increased Aβ phagocytosis in macrophages [89]. Poudel and colleagues demonstrated that a dendrimer composed of gallic acid and triethylene glycol (GATG), featuring 27 terminal morpholine groups ([G3]-Mor), can effectively reduce Aβ fibril formation [70]. In summary, dendrimers can improve cognitive, memory, and behavioral patterns, increase Aβ phagocytosis of macrophages, as well as lowering of Aβ fibril formation.

### Polymeric nanoparticles

**Fig. 9:**
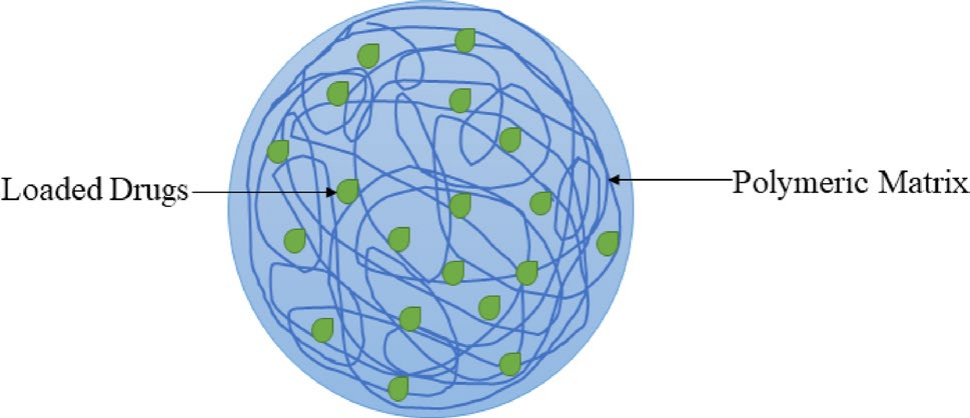
Structure of Polymeric Nanoparticles

Polymers arise as macromolecules made up of many repeating units that are systematic in a chain-like molecular architecture and can have a broad range of compositions, structures, and effects (Fig. 9). Polymers are used to develop nanoparticle systems optimized for specific biomedical applications due to their diverse compositions, structures, and properties. Polymeric nanoparticles are primarily employed in drug delivery, as well as in bioimaging and biosensing experiments [97]. The application of nanoparticles in medicine delivery has received more attention because of the growing significance of targeted delivery in drug delivery, which means that a lot of analysis has gone into developing polymeric nanoparticles that are systematic, tissue-specific, and most significantly, non-toxic. Numerous effective methods exist for preparing nanoparticles tailored for drug delivery, each varying based on the specific approach used to attach the medication to the nanoparticles. Nanoparticle-drug compounds can possess a capsule structure, such as polymeric nanoparticles or polymeric nanoconjugates; an amphiphilic core/shell structure, like polymeric micelles; or hyperbranched macromolecules with nanometer dimensions, referred to as dendrimers [98]. The principal restrictions are particle accumulation, polymer chemical reliability, raw materials utilised in production of nanoparticles, and the timely release of the active substance are critical to ensuring optimal results [99]. Polymeric nanoparticles can control the release of medicine by diffusing between the polymer matrix or degrading the matrix. Research has focused on their application as drug delivery systems for site-specific targeting of tumors and for facilitating transport of pharmaceuticals across biological barriers, particularly the BBB [100].

**Fig. 10:**
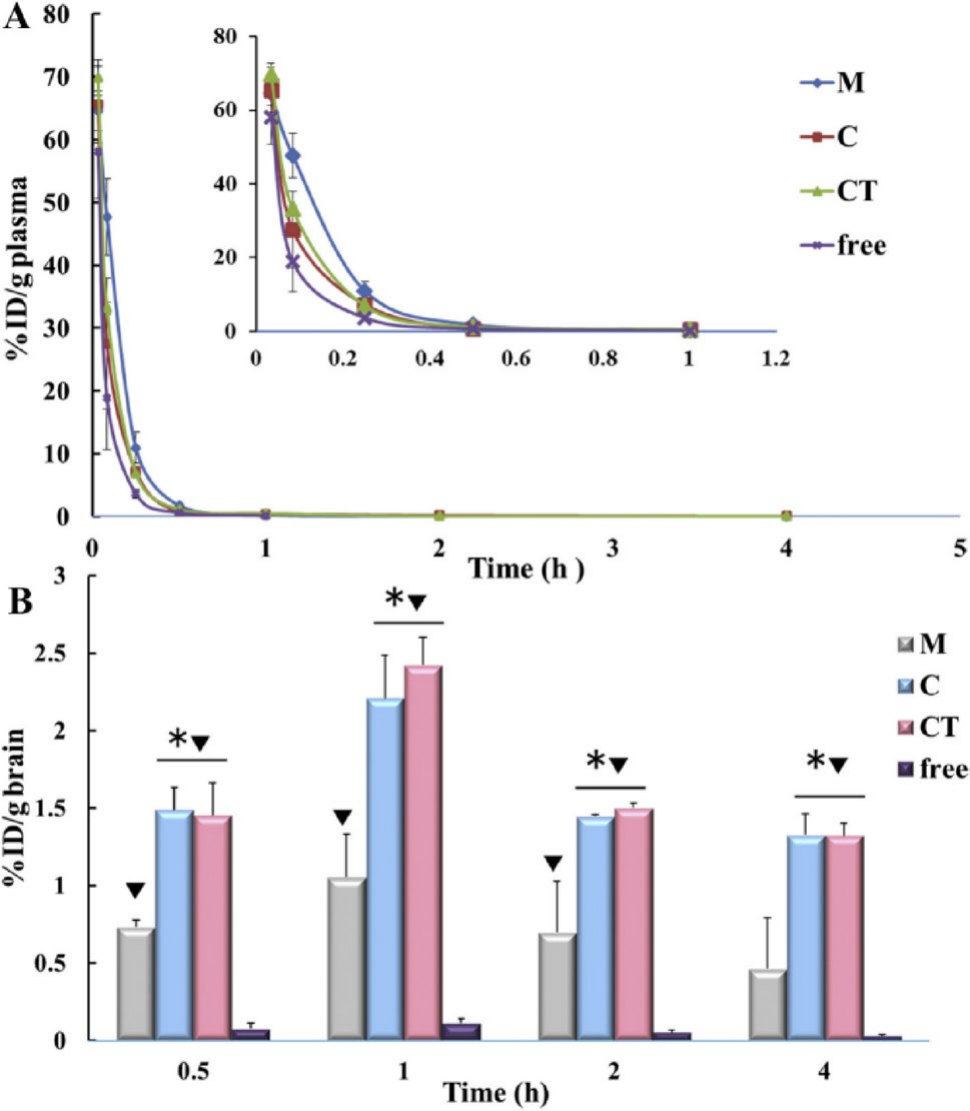
Pharmacokinetics results of PEGylated nanocomplexes demonstrate the concentration-time profiles of Cy3-siRNA in both plasma (**A**) and brain (**B**). Mice were administered M/Cy3-siRNA (M), C/Cy3-siRNA (**C**), CT/Cy3-siRNA (CT), and plain Cy3-siRNA (free) at a dosage of 32 µg/mouse. Statistically significant differences are indicated by *p < 0.05 when contrasted to the M group, and ▼*p* < 0.05 when contrasted to the free siRNA. Data are presented as Mean ± SD (*n* = 4). Reproduced with copyright permission from Elsevier [101]

Wang et al. developed a method to create specialized carriers for siRNA, a type of genetic material, for brain targeting [101]. The nanocomplexes (CT/siRNA) were made up of CGN- PEGylated poly(2-(*N*, *N*-dimethylamino) ethyl methacrylate) (PEG-PDMAEMA) and Tet1-PEG-PDMAEMA at a weight ratio of 1:1. These carriers were modified with CGN peptide to enhance their capacity to penetrate the BBB and Tet1 peptide to facilitate binding to neurons. The results showed a successful creation of a targeted gene delivery system that treats AD by targeting brain neurons. The biodistribution and pharmacokinetic studies exhibited that the half-lives of CT/Cy3-siRNA, C/Cy3-siRNA, and M/Cy3-siRNA in the blood were 0.97 h 1.00, and 1.01 h, importantly longer than that of pristine Cy3-siRNA (only 0.15 h) (Fig. 10A). Extended blood circulation of PEGylated nanocomplexes is advantageous for delivery of siRNA into brain. The maximum concentrations of CT/siRNA and C/siRNA were 2.42 ± 0.18%ID/g-brain and 2.21 ± 0.28%ID/g-brain, respectively, which were obviously higher than those of naked siRNA (0.11 ± 0.03%ID/g-brain) and M/siRNA (1.05 ± 0.18%ID/g-brain) (Fig. 10B). The nanocomplexes effectively penetrated the BBB and specifically delivered siRNA to neurons. The findings showed that CT/siRNA nanocomplexes have great potential for AD therapy [101]. According to Huo et al., PLGA nanospheres loaded with curcumin display potent stunt towards Aβ aggregation, making them a promising targeted drug delivery strategy for treating AD [102]. Yusuf et al. demonstrated that TQ-containing PLGA-NPs with polysorbate-80 (P-80) can safely and successfully facilitate the delivery of NPs across BBB and into the brain [103]. Huo et al. showed improved selenium NPs entangled in poly lactic-co-glycolic acid (PLGA) nanospheres for loading curcumin. The results displayed powerful inhibitory effects towards A aggregation in a transgenic AD mouse model, an attractive feature for a delivery system to treat AD [102].

Naki et al. designed polymer-drug conjugates comprising E)-N-(3-aminopropyl)cinnamide, memantine, and tacrine as good therapeutics for treating neurodegenerative disorders [103]. The findings from docking tests proved that the fusing of medicine within the polymers enhanced the anti-acetylcholinesterase activity [104]. Jeon et al.. report a study of vitamin D-loaded PLGA using a murine AD model [105]. They discovered a decrease in neuronal apoptosis with neuroinflammation, plus an increase in cognitive function [105]. According to Huo et al.., encapsulation of Selenium NPs into PLGA nanospheres with curcumin showed powerful prevention towards Aβ aggregation, suitable for the treatment of AD [102]. Yusuf and colleagues outlined that TQ-comprising PLGA-NPs with polysorbate-80 (P-80) might be safe plus effective in transporting NPs across the BBB [103].

In a study by Nanaki et al.., hybrid PNPs with poly(L-lactic acid) (PLA) and PLGA-based delivery systems were developed for expanded let out formulations that are able to lower dosing frequency and severe side effects [106]. Furthermore, they loaded PLA/PLGA hybrid NPs with galantamine, which exhibited prosperous nasal delivery in AD-induced rodent models [106]. Cano and colleagues proposed a binary drug therapy containing epigallocatechin-3-gallate (EGCG) and ascorbic acid (AA) in PLGA-PEG PNPs. In vitro and ex vivo experiments with EGCG/AA PNPs revealed that they interrupted tight junctions, making the BBB penetrable. Furthermore, in vivo studies in AD-induced mice showed lowered Aβ plaques plus neuroinflammation, improved memory, learning processes, and synaptogenesis [107].

Singh et al.. loaded galantamine into thiolated chitosan PNPs for intranasal administration. In vivo studies showed notable recuperation of amnesia-induced mice characterized by memory-augmenting impacts as well as the prevention of acetylcholinesterase activity Thiolated chitosan PNPs’ mucoadhesive properties enhanced their retention in the olfactory mucosa, resulting in improved permeation of galantamine by widening tight protein channels [108]. Mahl et al., showed that chitosan is an effectual chelating agent able to compete with Aβ, nor histidine for copper imperative [109]. A study by Liu et al.. showed enhanced organic NPs loaded with donepenzil through the introduction of regadenoson (Reg) at the end of PEG2000 [110]. Their aim was to improve drug delivery into brain and activate NO/cGMP plus PKG/PI3K signaling pathways. These findings revealed enhance learning and memory in Aβ-induced AD mice [110]. Polymeric nanoparticles are capable of inhibiting aggregation, treating neurodegenerative disorders, improving the anti-acetylcholinesterase effect, increasing memory, good transport across BBB via intranasal delivery, and capability of reducing Aβ plaques and neuroinflammation [100].

### Metal nanoparticles

**Fig. 11:**
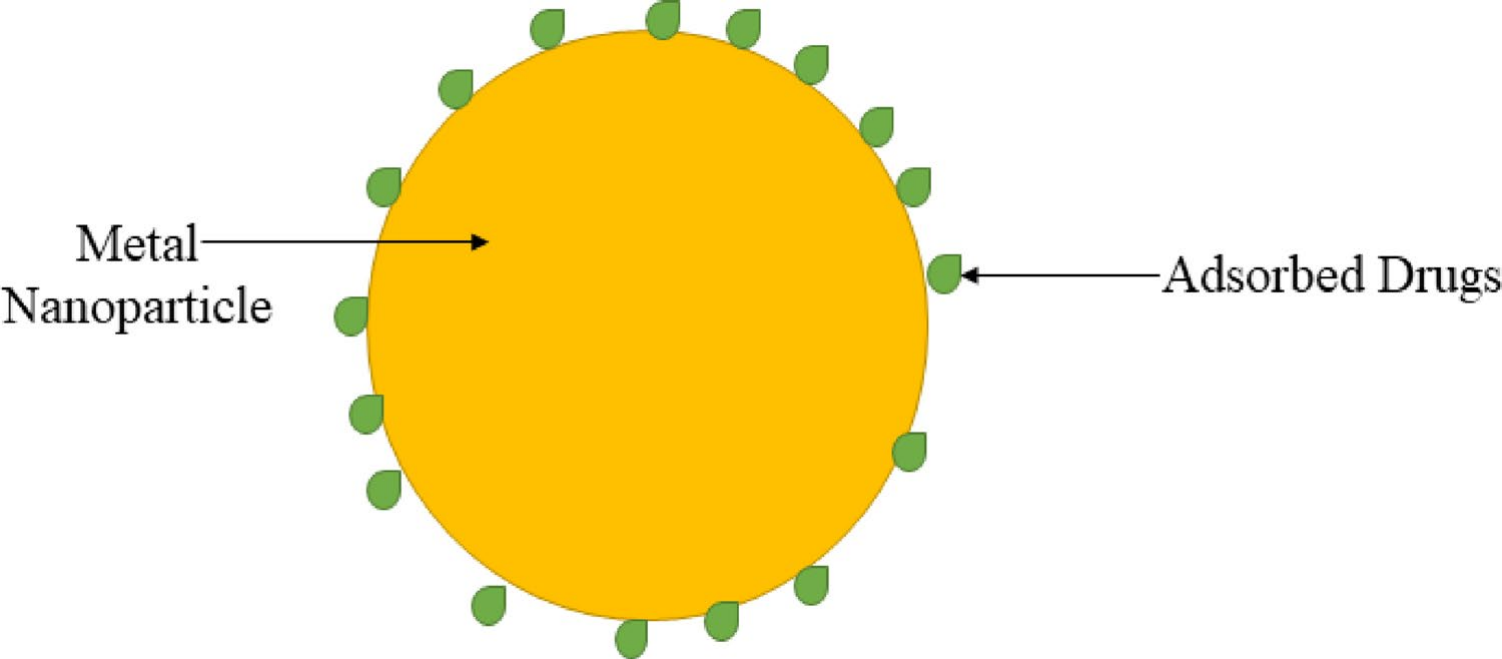
Structure of Metal Nanoparticles

Metal nanoparticle is suggested as transporters and therapeutic agents in the biomedical field due to their distinctive physicochemical effects and because of their physicochemical properties (Fig. 11). They can be used in a variety of biomedical fields. Metal nanoparticles can be formulated with different ligands to regulate their magnitude and formation, allowing them to be used in medicine delivery, diagnostics, and treating CNS diseases [111]. The benefits of noble metal-based nanoparticles, which are essential for medical implementation, include high biocompatibility, reliability, and the possibility of large-scale manufacturing while circumventing organic solvents, thus having a productive result on biological systems [112]. The effectiveness of nano delivery systems for brain targeting is contingent upon several critical factors, including nanoparticle size at the nanometric scale, surface charge, and morphology. However, the most pivotal aspect is the molecular recognition and interaction between a specific ligand conjugated to the surface of the nanoparticle and the molecule that is overexpressed at the intended site within the brain (active targeting) [113].

Gold nanoparticles (AuNPs) were investigated as powerful anti-Aβ therapeutics [114]. Hou synthesized 3.3 nm L- and D-glutathione stabilized gold nanoparticles. The finding shows that the NPs prevent accumulation of Aβ42 and cross the BBB when administered intravenously without significant toxicity. Moreover, the conjugation of nanoparticles with a chiral identification moiety shows a great therapeutic strategy for AD [114]. Santos and colleagues examined the effectiveness of AuNPs injected intracerebroventricularly with 100 µg of okadaic acid in an AD animal model [115]. The results showed that AuNPs without loaded drugs inhibited neuroinflammation, reinstated oxidant extents in the brain, including inhibited tau phosphorylation [115].

According to Sanati et al.., neuroprotective role of bare AuNPs was indicated via intraperitoneal routes and intrahippocampal in an AD-perspective rat model [115]. Findings showed an enhancement in neuronal survival, retention, and acquisition of learning and memory [116]. AuNP-based immunosensors are applied in the detection of tau and hallmarks of AD. According to Li et al.., formulation of anti-biofouling polymer polyethylene glycol-block-allyl glycidyl ether magnetic iron oxide nanoparticles for the discovery of Aβ peptides and tau protein in AD [117]. The findings indicated that this formulation can put an end to non-specific interactions with Aβ peptides plus tau proteins [117]. The reports on AuNPs show that they can prevent neuroinflammation, aggregation of Aβ42, tau phosphorylation. AuNPs are capable of crossing BBB, show good neuronal survival, and retention, and improve memory, and lastly can stop non-particular interactivity with Aβ peptides and tau proteins.

### Liposome nanoparticles

**Fig. 12:**
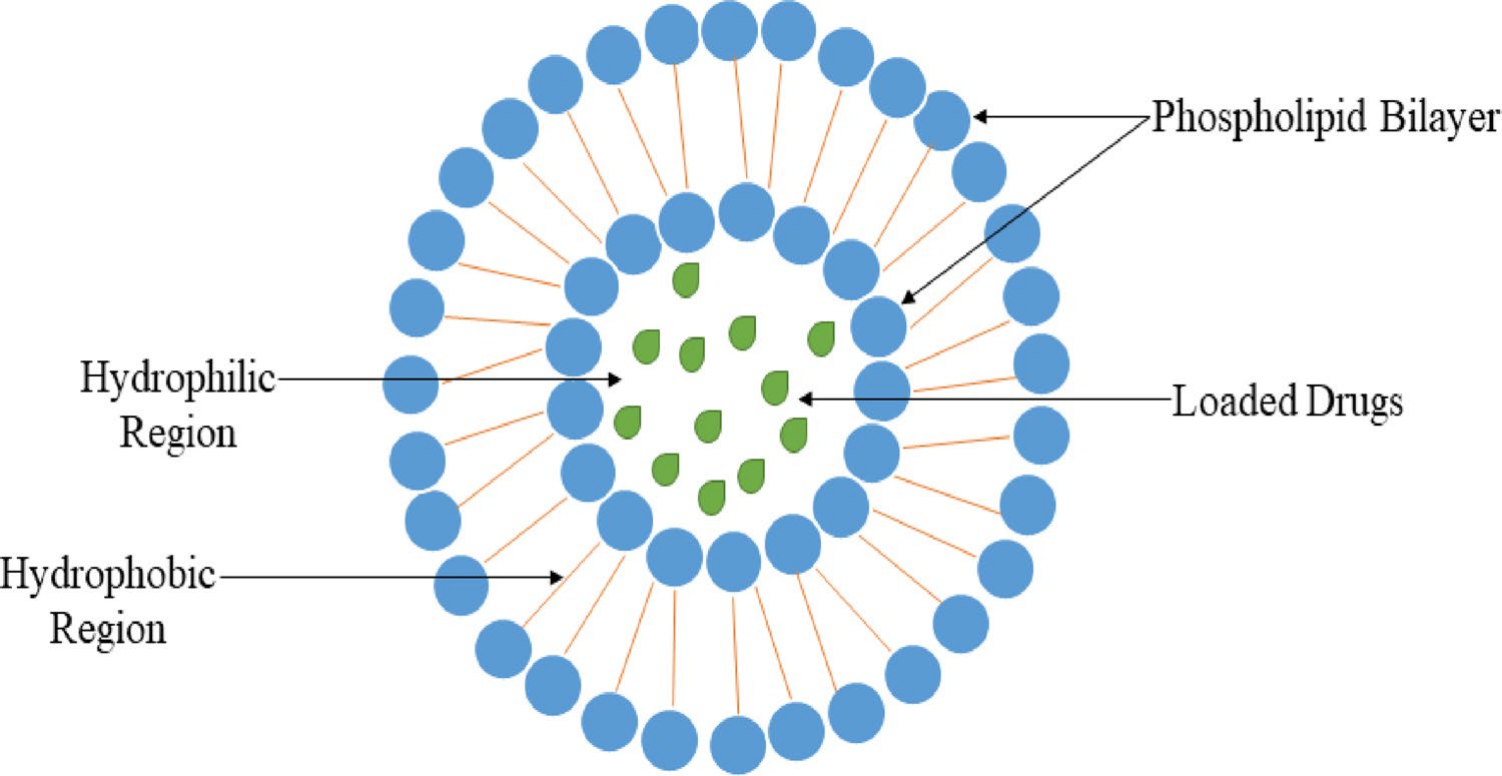
Structure of Liposome Nanoparticles

Liposomes are functional as drug transporters (Fig. 12), but they need complex manufacture procedure involving organic solvents, have less drug entrapment regulation, and are strenuous to execute on a large scale [118]. The absolute attribute of the liposomal creation include homogeneous particle magnitude (102 ± 3.3) and low PI (0.28 ± 0.03), smaller magnitude of vesicles having ability to ease the immersion of the medicine; (ii) the system has a higher encapsulation efficiency (EE) of 84.91 ± 3.31%, which may lead to improved bioavailability of the drug; (iii) Additionally, the results from in vitro drug release studies indicated a good distribution of drug particles, which enhances the absorption of DNP from the nasal cavity [119].

Regina et al. designed Curcumin-loaded liposomes, which are capable of delivering drugs to CNS, crossing BBB, and showing an anti-Alzheimer impact [120]. Samudre et al. prepared a curcumin liposomal formulation with mucoadhesive properties for intranasal administration. The in vivo study evaluated bioavailability of the medicine in brain following intranasal administration, suggesting that xanthan gum-coated Curcumin liposomes represent an innovative and effective drug delivery system specifically designed for targeting brain through the nasal pathway [121]. Li and colleagues studied the reaction of intranasal administration of galantamine-loaded flexible liposomes with the capability to inhibit the regulation of acetylcholinesterase, as well as the pharmacokinetic behavior of galantamine (GH). It has been shown that regulation of acetylcholinesterase stunt of GH was greatly improved by nasal administration, as contrasted to oral one, with special regard to GH stacked in flexible liposomes [122].

Fernandes et al.. prepared Curcumin-loaded liposomes, which were not cytotoxic and lowered oxidative stress induced in SH-SY5Y neuronal cells [123]. These attributes arise as useful for neuroprotection [123]. Hernandez et al.. formulated liposomes loaded with mAPO and phosphatidic acid. Findings present phosphatidic acid with increased affinity against binding of Aβ, whereas mAPO improves penetration of BBB [124]. Santos shows the loading of Apolipoprotein E2 with liposome, and the results demonstrated a therapeutic impact in AD via gene adaptation [125]. Moreover, ApoE2 loaded to intended brain tissue exhibited treatment advantage in AD [124]. Andrede et al.. reported a conjugation of Tf to liposomes’ exterior to direct CA-loaded nanoparticles to BBB [126]. Conjugation exhibited fit encapsulation, regulation, and physical stability for 2 months. Moreover, CA-loaded Tf-functionalized liposomes can inhibit Aβ accumulation, and fibril formation as well as disaggregate mature fibrils [125].

**Fig. 13:**
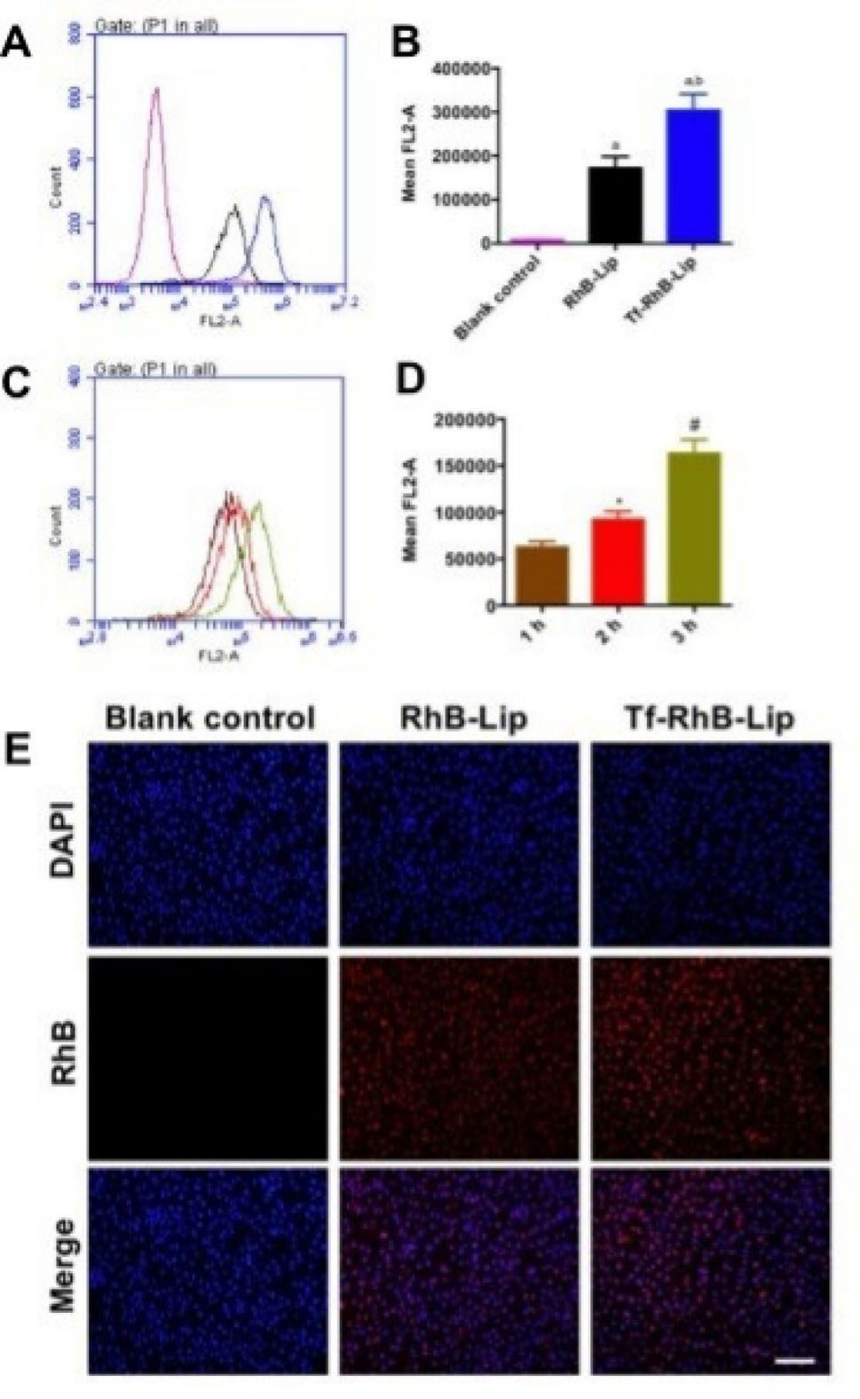
Cellular Uptake and Dispersal Following Incubation with Various Liposomal Formulations. (**A**) Cellular uptake in hCMEC/D3 cells treated with transferrin-modified PEGylated liposomes compared to plain RhB liposomes; (**B**) Quantitative analysis of fluorescence intensity is presented as mean ± SD (*n* = 3). Statistically significant differences are indicated as follows: a, vs. Blank control; b, vs. RhB liposomes; *P* < 0.05. (**C**) Cellular uptake dynamics of hCMEC/D3 cells treated with transferrin-altered PEGylated liposomes over different time points; (**D**) Quantitative analysis of fluorescence intensity is again presented as mean ± SD (*n* = 3), with significant differences noted as follows: *, vs. 1 h; #, vs. 2 h; *P* < 0.05. (**E**) Analysis of fluorescence intensity in hCMEC/D3 cells cultured with the various formulations, as observed through fluorescence microscopy (scale bar = 100 μm, *n* = 3) [131]

Saffari and colleagues loaded phosphatidylserine-based liposomes with metformins, that exhibited higher effectiveness in enhancing memory plus studying deficits as well as lowering neuroinflammation in AD-induced rat models compared to metformin [127]. Kong et al.. found that transferrin-modified PEGylated liposomes enabled osthole to cross the blood-brain barrier and accumulate in the brain, thereby alleviating AD-related pathology in APP/PS-1 mice [128]. The in vitro cellular Uptake and Targeting studies significantly showed that the fluorescence strength of transferrin-modified PEGylated liposomes was 1.61-fold greater than that of unmodified, suggesting that transferrin-modified liposomes possessed better penetrability in human blood-brain barrier (hCMEC/D3) cells (Fig. 13A and B). The studies indicate that the cellular uptake of transferrin-modified liposomes increased progressively with longer incubation times (Fig. 13C and D). Fluorescence microscopy (Fig. 13E) images clearly exhibited red fluorescence in transferrin-modified PEGylated liposomes and were much stronger than that observed in plain PEGylated liposomes, revealing that drug accumulation in the transferrin-modified PEGylated liposomes group was higher in the hCMEC/D3 cells [128].

Kocsis and colleagues formulated several dye sections on the exterior of a vesicle to increase the linking of empathy for α-synuclein fibrils to improve the identification of amyloid accumulates [129]. Yang and colleagues successfully loaded neuronal mitochondria-targeted micelles with resveratrol [130]. Establishing a dynamic balance between mitochondrial fusion and fission is essential for restoring cognitive performance in APP/PS1 transgenic AD mice [130]. According to Agwa et al.. lactoferrin fused with linoleic acid micelles exhibited increased cognitive capabilities, biodistribution in the brain, reduced brain oxidative stress, apoptosis, acetylcholine esterase activity, and inflammation [131]. Reports on liposomes show that they possess an ability to reduce oxidative stress, neuroinflammation, apoptosis, acetylcholine esterase activity, increased affinity against binding of Aβ and penetration in BBB, therapeutic impact towards AD, improvement memory, and identification of amyloid aggregation.

**Table 1:** An overview of nanoformulated drugs for alzheimer’s disease (2013–2023)

Drug	Nanoformulation Type	Administration	Key Findings	Reference
Clozapine	SNL’s	Intravenous and intraduodenal routes	Effectively managed AD	[61]
Donepezil	SNL’s	Nasal route	Improved release rate and transportation	[64]
Ferulic acid	SNL’S	Nasal mucosal adhesion	Enhanced memory functions	[71]
Revastigmine	NCL’s	Intranasal path	Persuaded fast drug release	[77]
Resveratrol (RV)	NLC’S	Nasal route	Reduced inflammation	[78]
Curcumin and donepezil	NLC’S	Nasal path	Improved mental abilities	[79]
Chitosan	NLC’s	Intranasal path	Enhanced safe drug delivery	[80]
Memantine	NE	Intranasal route	Moderated critical AD for cognition	[87]
Glycerol and labrasol	NE	Intranasal route	enhanced drug absorption	[88]
Curcumin	liposomal formulation	Intranasal path	Enhanced drug absorption	[121]

## Clinical studies for the treatment of AD

The current phases, along with a list of drugs that are undergoing clinical studies for AD trials, are presented in Table 2. In a transgenic mouse model designed for AD, aducanumab has exhibit the ability to cross the BBB, attach to parenchymal Aβ, and reduce both soluble and insoluble Aβ levels in a dose-dependent manner. The decrease was observed and associated with a deceleration in the progression of clinical deterioration, as evaluated by the Clinical Dementia Rating—Sum of Boxes and the Mini-Mental State Examination scores. Should the ongoing phase 3 clinical trials demonstrate a reduction in clinical decline, it would bolster the amyloid hypothesis [132]. Crenezumab is currently being studied in the CREAD trials, which focus on evaluating its effectiveness and safety as contrasted to a placebo in participants with prodromal to mild AD. Moreover, elenbecestat, which is a BACE-1 stunt, is another treatment being investigated in this area. A phase 2b trial of elenbecestat in amyloid-PET-positive individuals with mild cognitive impairment (MCI), prodromal AD, or mild AD showed a dose-dependent reduction in cerebrospinal fluid (CSF) Aβ levels; however, there were no significant improvements in the AD Composite Score or the Clinical Dementia Rating Sum of Boxes (CDR-SB) score [133]. A phase 4 trial (NCT05004987) began in February 2022 to explore if there is a decrease in depressive symptoms from escitalopram oxalate is linked to the normalization of AD biomarkers in cerebrospinal fluid (CSF) and inflammatory markers in peripheral blood. The main outcome measures include alters in levels of CSF Aβ40 and Aβ42, markers of vascular dysfunction, and scores on the Montgomery–Asberg Depression Rating Scale at the end of week 8 [134]. NE3107 is an anti-inflammatory insulin sensitizer that can cross the BBB, bind to ERK and modulate AD pathology by selectively preventing inflammation. This compound effectively targets inflammation-driven mediators controlled by ERK and NF-κB, such as TNF-α without disrupting their normal cellular functions [134]. The rise of tau oligomers in the brains and CSF of individuals with AD has prompted the development of drugs aimed at preventing tau aggregation or breaking down existing aggregates. Despite their promise, treatments like methylthioninium chloride and its second-generation derivatives, such as TRx0237, have not shown effectiveness in clinical trials. A Phase II study involving TRx0237 was halted after just a few months due to “administrative” issues. Meanwhile, the Phase III studies have shown disappointing results concerning cognitive improvement. Nevertheless, it remains unclear whether these drugs effectively inhibit tau aggregation in humans [132, 135].

**Table 2:** List of drugs that are currently in clinical studies for the treatment of AD

Drug	Mechanism of action	Therapeutic purpose	Stage of AD	Target	Phase	Reference
Aducanumab	Disrupts formation of Aβ plaques and oligomers.	N, N Dimethyltryptamine (DMT)	Mild to moderate	Amyloid	3	[132]
Crenezumab	Monoclonal antibody directed at oligomers	Remove amyloid	Mild	Remove amyloid	3	[133]
LY3372689	A potent inhibitor of tau protein aggregation.	DMT	Moderate	Tau	2	[132]
Escitalopram Oxalate	Selective serotonin reuptake inhibitor	Neuropsychiatric symptoms	--------	Depression	4	[134]
Hydralazin5.e	Antioxidant	DMT	Mild to moderate	Oxidative stress	3	[132]
NE3107	anti-inflammatory insulin sensitizer that effectively binds to ERK.	Anti-inflammatory	-------	Anti-inflammatory	2	[134]
TRx0237 (21)	inhibitor of nitric oxide synthase and guanylate cyclase.	Tau aggregation inhibitor	-------	Tau aggregation inhibitor	3	[135]

## Conclusion

AD progresses slowly, making it challenging to demonstrate the effectiveness of treatments in preventing or slowing its progression [136]. Only a small percentage of potential participants consider participating in Alzheimer’s clinical trials. Overcoming participation barriers and involving more people in Alzheimer’s clinical trials is crucial for developing effective prevention and treatment strategies. A survey and interviews with nearly 900 AD stakeholders were conducted to better understand these barriers and devise a strategy for overcoming them [137]. Nanotechnology has great potential in treating AD but faces challenges such as delivering nanotherapeutics across the blood-brain barrier and the potential toxicity of nanoparticles. Overcoming these challenges is crucial for its success in the clinic [138]. The current limitations of existing therapies and translational challenges in applying nanotechnology-based approaches in AD treatment include BBB which limits drug delivery to the brain, limited efficacy, and multifactorial nature of AD, making it strenuous to target specific mechanisms [139]. There is a significant effort required to treat AD, including delivering the appropriate pharmaceuticals to the correct neurons and finding suitable formulations. Most of these nanomedicines exhibit promising results, and a lot has been discovered from polymeric nanoparticles, which have shown promising strategies. There are significant gaps and questions that remain regarding use of NPs in the treatment of neuro disorders. Another issue is understanding which route of administration is appropriate for nanomedicine. The complex pathology of AD makes it challenging to design nano-formulations that can reach the affected brain regions effectively to deliver the therapeutic agents precisely. Persistent knowledge gaps highlight the critical need for innovative research into effective therapeutics to address AD unmet clinical demands. To effectively research AD, a combination of study types is required such as clinical validation, BBB-penetration, long-term safety or transcriptomic analyses.

## Data Availability

No datasets were generated or analysed during the current study.

## References

[1] Gholami A Alzheimer’s disease: the role of proteins in formation, mechanisms, and new therapeutic approaches. Neurosci Lett .2023;817. 10.1016/j.neulet.2023.137532 10.1016/j.neulet.2023.137532 37866702

[2] Fu D, Liu D, Zhang L, Sun L (2020) Self-assembled fluorescent tripeptide nanoparticles for bioimaging and drug delivery applications. Chin Chem Lett 31. 10.1016/j.cclet.2020.07.011

[3] Shamrat FMJM, Akter S, Azam S, Karim A, Ghosh P, Tasnim Z et al (2023) AlzheimerNet: an effective deep learning based proposition for alzheimer’s disease stages classification from functional brain changes in magnetic resonance images. IEEE Access. 10.1109/access.2023.3244952

[4] Watt AD, Jenkins NL, McColl G, Collins S, Desmond PM (2019) Ethical issues in the treatment of late-stage alzheimer’s disease. J Alzheimer’s Dis 68. 10.3233/jad-180865 10.3233/JAD-180865 PMC6484269 30475773

[5] Gustavsson A, Norton N, Fast T, Frölich L, Georges J, Holzapfel D et al (2023) Global estimates on the number of persons across the alzheimer’s disease continuum. Alzheimer’s Dement 19. 10.1002/alz.12694 10.1002/alz.12694 35652476

[6] Knopman DS, Amieva H, Petersen RC, Chételat G, Holtzman DM, Hyman BT et al (2021) Alzheimer Disease Nat Rev Dis Primers 7. 10.1038/s41572-021-00269-y 10.1038/s41572-021-00269-y PMC8574196 33986301

[7] De-Paula VJ, Radanovic M, Diniz BS, Forlenza OV (2012) Alzheimer’s disease. Subcell Biochem. 10.1007/978-94-007-5416-4_14 23225010 10.1007/978-94-007-5416-4_14

[8] Armstrong RA (2019) Risk factors for alzheimer’s disease. Folia Neuropathol 57. 10.5114/fn.2019.85929 10.5114/fn.2019.85929 31556570

[9] Anand P, Singh B (2013) A review on cholinesterase inhibitors for alzheimer’s disease. Arch Pharm Res. 10.1007/s12272-013-0036-3 23435942 10.1007/s12272-013-0036-3

[10] Breijyeh Z, Karaman R (2020) Comprehensive review on alzheimer’s disease: causes and treatment. Molecules. 10.3390/molecules25245789 33302541 10.3390/molecules25245789 PMC7764106

[11] Eldufani J, Blaise G (2019) The role of acetylcholinesterase inhibitors such as neostigmine and Rivastigmine on chronic pain and cognitive function in aging: A review of recent clinical applications. Alzheimer’s Dement Transl Res Clin Interv 5. 10.1016/j.trci.2019.03.004 10.1016/j.trci.2019.03.004 PMC6551376 31194017

[12] Sharma K (2019) Cholinesterase inhibitors as alzheimer’s therapeutics. Mol Med Rep 20. 10.3892/mmr.2019.10374 10.3892/mmr.2019.10374 PMC6625431 31257471

[13] Zhang Y, Geng R, Liu M, Deng S, Ding J, Zhong H et al (2023) Shared peripheral blood biomarkers for alzheimer’s disease, major depressive disorder, and type 2 diabetes and cognitive risk factor analysis. Heliyon 9. 10.1016/j.heliyon.2023.e14653 10.1016/j.heliyon.2023.e14653 PMC10040717 36994393

[14] Pham DT, Tiyaboonchai W (2020) Fibroin nanoparticles: A promising drug delivery system. Drug Deliv. 10.1080/10717544.2020.1736208. 27 32157919 10.1080/10717544.2020.1736208 PMC7144220

[15] Hsu CY, Rheima AM, Kadhim MM, Ahmed NN, Mohammed SH, Abbas FH et al (2023) An overview of nanoparticles in drug delivery: properties and applications. South Afr J Chem Eng 46. 10.1016/j.sajce.2023.08.009

[16] Fonseca-Santos B, Gremião MPD, Chorilli M (2015) Nanotechnology-based drug delivery systems for the treatment of alzheimer’s disease. Int J Nanomed 10. 10.2147/IJN.S87148 10.2147/IJN.S87148 PMC4531021 26345528

[17] Gomes MJ, das Neves J, Sarmento B (2014) Nanoparticle-based drug delivery to improve the efficacy of antiretroviral therapy in the central nervous system. Int J Nanomed 9. 10.2147/IJN.S45886 10.2147/IJN.S45886 PMC3984056 24741312

[18] Satalkar P, Elger BS, Hunziker P, Shaw D (2016) Challenges of clinical translation in nanomedicine: A qualitative study. Nanomed Nanatechnol Biol Med 12. 10.1016/j.nano.2015.12.376 10.1016/j.nano.2015.12.376 26772431

[19] Pardo-Moreno T, González-Acedo A, Rivas-Domínguez A, García-Morales V, García-Cozar FJ, Ramos-Rodríguez JJ et al (2022) Therapeutic approach to alzheimer’s disease: current treatments and new perspectives. Pharmaceutics 14. 10.3390/pharmaceutics14061117 10.3390/pharmaceutics14061117 PMC9228613 35745693

[20] Larkin HD (2022) First donepezil transdermal patch approved for alzheimer disease. JAMA 327. 10.1001/jama.2022.6662 10.1001/jama.2022.6662 35503362

[21] Yiannopoulou KG, Papageorgiou SG (2020) Current and future treatments in alzheimer disease: an update. J Cent Nerv Syst Dis 12. 10.1177/1179573520907397 10.1177/1179573520907397 PMC7050025 32165850

[22] Kabir MT, Sufian MA, Uddin MS, Begum M, Akhter S, Islam A et al (2019) NMDA receptor antagonists: repositioning of memantine as a multitargeting agent for alzheimer’s therapy. Curr Pharm Des 25. 10.2174/1381612825666191011102444 10.2174/1381612825666191011102444 31604413

[23] Matsunaga S, Kishi T, Nomura I, Sakuma K, Okuya M, Ikuta T et al (2018) The efficacy and safety of memantine for the treatment of alzheimer’s disease. Expert Opin Drug Saf 17. 10.1080/14740338.2018.1524870 10.1080/14740338.2018.1524870 30222469

[24] Rogawski MA, Wenk GL (2003) The neuropharmacological basis for the use of memantine in the treatment of alzheimer’s disease. CNS Drug Rev. 10.1111/j.1527-3458.2003.tb00254.x. 9 14530799 10.1111/j.1527-3458.2003.tb00254.x PMC6741669

[25] Lipton SA (2005) The molecular basis of memantine action in alzheimer’s disease and other neurologic disorders: low-affinity, uncompetitive antagonism. Curr Alzheimer Res 2. 10.2174/1567205053585846 10.2174/1567205053585846 15974913

[26] Adlimoghaddam A, Neuendorff M, Roy B, Albensi BC (2018) A review of clinical treatment considerations of donepezil in severe Alzheimer’s disease. CNS Neurosci Ther. 10.1111/cns.13035 30058285 10.1111/cns.13035 PMC6489741

[27] Hong YJ, Han HJ, Youn YC, Park KW, Yang DW, Kim S et al (2019) Safety and tolerability of donepezil 23 mg with or without intermediate dose titration in patients with Alzheimer’s disease taking donepezil 10 mg: a multicenter, randomized, open-label, parallel-design, three-arm, prospective trial. Alzheimers Res Ther 11. 10.1186/s13195-019-0492-1 10.1186/s13195-019-0492-1 PMC6492390 31039806

[28] Kumar A, Sidhu J, Goyal A, Tsao JW, Doerr C (2021) Alzheimer disease (nursing).Stat Pearls

[29] Kalola UK, Nguyen H (2021) Galantamine. Stat Pearls

[30] Janssen B, Schäfer B, Galantamine (2017) ChemTexts. 10.1007/s40828-017-0043-y

[31] Lilienfeld S (2002) Galantamine—a novel cholinergic drug with a unique dual mode of action for the treatment of patients with alzheimer’s disease. CNS Drug Rev. 10.1111/j.1527-3458.2002.tb00221.x 12177686 10.1111/j.1527-3458.2002.tb00221.x PMC6741688

[32] Kastanenka KV, Bussiere T, Shakerdge N, Qian F, Weinreb PH, Rhodes K et al (2016) Immunotherapy with aducanumab restores calcium homeostasis in Tg2576 mice. J Neurosci. 10.1523/JNEUROSCI.2080-16.2016 27810931 10.1523/JNEUROSCI.2080-16.2016 PMC5157102

[33] Rahman A, Hossen MA, Chowdhury MFI, Bari S, Tamanna N, Sultana SS et al (2023) Aducanumab for the treatment of alzheimer’s disease: a systematic review. Psychogeriatrics 23. 10.1111/psyg.12944 10.1111/psyg.12944 PMC11578022 36775284

[34] Boris D, Fadel B, Pope ED III, Shi J, Mari Z, Sabbagh MN (2021) Critical appraisal of amyloid lowering agents in AD. Curr Neurol Neurosci Rep. 10.1007/s11910-021-01125-y 10.1007/s11910-021-01125-y PMC8192384 34110536

[35] Arndt JW, Qian F, Smith BA, Quan C, Kilambi KP, Bush MW et al (2018) Structural and kinetic basis for the selectivity of aducanumab for aggregated forms of amyloid-β. Sci Rep 8. 10.1038/s41598-018-24501-0 10.1038/s41598-018-24501-0 PMC5913127 29686315

[36] Guo H, Wang G, Zhai Z, Huang J, Huang Z, Zhou Y et al (2024) Rivastigmine nasal spray for the treatment of alzheimer’s disease: olfactory deposition and brain delivery. Int J Pharm 652. 10.1016/j.ijpharm.2024.123809 10.1016/j.ijpharm.2024.123809 38224760

[37] Birks JS, Evans JG (2015) Rivastigmine for alzheimer’s disease. Cochrane Database Syst Rev. 10.1002/14651858.CD001191.pub4 25858345 10.1002/14651858.CD001191.pub3

[38] Müller T (2007) Rivastigmine in the treatment of patients with alzheimer’s disease. Neuropsychiatr Dis Treat. 10.2147/nedt.2007.3.2.211 19300554 10.2147/nedt.2007.3.2.211 PMC2654625

[39] Takahashi JA, Sande D, da Silva Lima G, e Moura MAF, Lima MTNS (2019) Fungal metabolites as promising new drug leads for the treatment of alzheimer’s disease. Stud Nat Prod Chem 62. 10.1016/B978-0-444-64185-4.00001-0

[40] Abbott NJ (2013) Blood–brain barrier structure and function and the challenges for CNS drug delivery. J Inherit Metab Dis. 10.1007/s10545-013-9608-0 23609350 10.1007/s10545-013-9608-0

[41] Daneman R, Prat A (2015) The blood–brain barrier. Cold Spring Harb Perspect Biol. 10.1101/cshperspect.a020412 25561720 10.1101/cshperspect.a020412 PMC4292164

[42] Wu D, Chen Q, Chen X, Han F, Chen Z, Wang Y The blood–brain barrier: structure, regulation, and drug delivery. 8, Signal Transduction and Targeted Therapy. Springer Nature.2023;8. 10.1038/s41392-023-01481-w 10.1038/s41392-023-01481-w PMC10212980 37231000

[43] Pandit R, Chen L, Götz J (2020) The blood-brain barrier: physiology and strategies for drug delivery. Adv Drug Deliv Rev. 10.1016/j.addr.2019.11.009 31790711 10.1016/j.addr.2019.11.009

[44] Erickson MA, Banks WA (2013) Blood–brain barrier dysfunction as a cause and consequence of Alzheimer’s disease. J Cereb Blood Flow Metab. 10.1038/jcbfm.2013.135 23921899 10.1038/jcbfm.2013.135 PMC3790938

[45] Banks WA (2012) Drug delivery to the brain in Alzheimer’s disease: consideration of the blood–brain barrier. Adv Drug Deliv Rev. 10.1016/j.addr.2011.12.005 22202501 10.1016/j.addr.2011.12.005 PMC3389492

[46] Luo S, Ma C, Zhu MQ, Ju WN, Yang Y, Wang X (2020) Application of iron oxide nanoparticles in the diagnosis and treatment of neurodegenerative diseases with emphasis on Alzheimer’s disease. Front Cell Neurosci. 10.3389/fncel.2020.00021 32184709 10.3389/fncel.2020.00021 PMC7058693

[47] Madara JL (1998) Regulation of the movement of solutes across tight junctions. Annu Rev Physiol 60. 10.1146/annurev.physiol.60.1.143 10.1146/annurev.physiol.60.1.143 9558458

[48] Martinez MN, Amidon GL (2002) A mechanistic approach to understanding the factors affecting drug absorption: a review of fundamentals. J Clin Pharmacol. 10.1177/00970002042006005 12043951 10.1177/00970002042006005

[49] Bickel U (1995) Antibody delivery through the blood-brain barrier. Adv Drug Deliv Rev. 15.https://https://doi.org/10.1016/0169-409X(95)00005-R 35524390

[50] Hayes GR, Enns CA, Lucas JJ (1992) Identification of the O-linked glycosylation site of the human transferrin receptor. Glycobiology. 10.1093/glycob/2.4.355 1421757 10.1093/glycob/2.4.355

[51] Kadry H, Noorani B, Cucullo L (2020) A blood–brain barrier overview on structure, function, impairment, and biomarkers of integrity. Fluids Barriers CNS. 10.1186/s12987-020-00230-3 33208141 10.1186/s12987-020-00230-3 PMC7672931

[52] Haqqani AS, Thom G, Burrell M, Delaney CE, Brunette E, Baumann E et al (2018) Intracellular sorting and transcytosis of the rat transferrin receptor antibody OX26 across the blood–brain barrier in vitro is dependent on its binding affinity. J Neurochem. 10.1111/jnc.14482 29877588 10.1111/jnc.14482 PMC6175443

[53] Alberts B (2002) Molecular biology of the cell 4th edition. (No Title)

[54] Teixidó M, Zurita E, Malakoutikhah M, Tarragó T, Giralt E (2007) Diketopiperazines as a tool for the study of transport across the blood – brain barrier (BBB) and their potential use as BBB-shuttles. J Am Chem Soc. 10.1021/ja073522o 17764181 10.1021/ja073522o

[55] Virgone-Carlotta A, Dufour E, Bacot S, Ahmadi M, Cornou M, Moni L et al (2016) New diketopiperazines as vectors for peptide protection and brain delivery: synthesis and biological evaluation. J Label Compd Radiopharm. 10.1002/jlcr.3442 10.1002/jlcr.3442 27611733

[56] Kashyap K, Shukla R (2019) Drug delivery and targeting to the brain through nasal route: mechanisms, applications and challenges. Curr Drug Deliv. 10.2174/1567201816666191029122740 31660815 10.2174/1567201816666191029122740

[57] Sawant KK, Dodiya SS (2008) Recent advances and patents on solid lipid nanoparticles. Recent Pat Drug Deliv Formul. 10.2174/187221108784534081 19075903 10.2174/187221108784534081

[58] Fenton OS, Olafson KN, Pillai PS, Mitchell MJ, Langer R (2018) Advances in biomaterials for drug delivery. Adv Mater. 10.1002/adma.201705328 29736981 10.1002/adma.201705328 PMC6261797

[59] Müller RH, Radtke M, Wissing SA (2002) Solid lipid nanoparticles (SLN) and nanostructured lipid carriers (NLC) in cosmetic and dermatological preparations. Adv Drug Deliv Rev. 10.1016/s0169-409x(02)00118-7 12460720 10.1016/s0169-409x(02)00118-7

[60] Stanisic D, Costa AF, Cruz G, Durán N, Tasic L (2018) Applications of flavonoids, with an emphasis on hesperidin, as anticancer prodrugs: phytotherapy as an alternative to chemotherapy. Stud Nat Prod Chem. 10.1016/B978-0-444-64056-7.00006-4

[61] Naser SS, Singh D, Preetam S, Kishore S, Kumar L, Nandi A et al (2023) Posterity of nanoscience as lipid nanosystems for alzheimer’s disease regression. Mater Today Bio. 10.1016/j.mtbio.2023.100701 37415846 10.1016/j.mtbio.2023.100701 PMC10320624

[62] Campisi A, Sposito G, Pellitteri R, Santonocito D, Bisicchia J, Raciti G et al (2022) Effect of unloaded and curcumin-loaded solid lipid nanoparticles on tissue transglutaminase isoforms expression levels in an experimental model of Alzheimer’s disease. Antioxidants. 10.3390/antiox11101863 36290586 10.3390/antiox11101863 PMC9599010

[63] Mir Najib Ullah SN, Afzal O, Altamimi ASA, Ather H, Sultana S, Almalki WH et al (2023) Nanomedicine in the management of alzheimer’s disease: State-of-the-art. Biomedicines 11. 10.3390/biomedicines11061752 10.3390/biomedicines11061752 PMC10296528 37371847

[64] Yasir M, Chauhan I, Haji MJ, Tura AJ, Saxena PK (2018) Formulation and evaluation of glyceryl behenate based solid lipid nanoparticles for the delivery of donepezil to brain through nasal route. Res J Pharm Technol. 10.5958/0974-360X.2018.00523.1

[65] Topal GR, Mészáros M, Porkoláb G, Szecskó A, Polgár TF, Siklós L et al (2020) ApoE-targeting increases the transfer of solid lipid nanoparticles with donepezil cargo across a culture model of the blood–brain barrier. Pharmaceutics 10.3390/pharmaceutics13010038 33383743 10.3390/pharmaceutics13010038 PMC7824445

[66] Dara T, Vatanara A, Sharifzadeh M, Khani S, Vakilinezhad MA, Vakhshiteh F et al (2019) Improvement of memory deficits in the rat model of alzheimer’s disease by erythropoietin-loaded solid lipid nanoparticles. Neurobiol Learn Mem. 10.1016/j.nlm.2019.107082 31493483 10.1016/j.nlm.2019.107082

[67] Chauhan MK, Sharma PK (2019) Optimization and characterization of Rivastigmine nanolipid carrier loaded transdermal patches for the treatment of dementia. Chem Phys Lipids. 10.1016/j.chemphyslip.2019.104794 31361985 10.1016/j.chemphyslip.2019.104794

[68] Mendes IT, Ruela ALM, Carvalho FC, Freitas JTJ, Bonfilio R, Pereira GR (2019) Development and characterization of nanostructured lipid carrier-based gels for the transdermal delivery of donepezil. Colloids Surf B Biointerfaces. 10.1016/j.colsurfb.2019.02.007 30763792 10.1016/j.colsurfb.2019.02.007

[69] Pinheiro RGR, Granja A, Loureiro JA, Pereira MC, Pinheiro M, Neves AR et al (2020) Quercetin lipid nanoparticles functionalized with transferrin for alzheimer’s disease. Eur J Pharm Sci. 10.1016/j.ejps.2020.105314 32200044 10.1016/j.ejps.2020.105314

[70] Poudel P, Park S (2022) Recent advances in the treatment of alzheimer’s disease using nanoparticle-based drug delivery systems. Pharmaceutics. 10.3390/pharmaceutics14040835 35456671 10.3390/pharmaceutics14040835 PMC9026997

[71] Hu L, Tao Y, Jiang Y, Qin F (2023) Recent progress of nanomedicine in the treatment of Alzheimer’s disease. Front Cell Dev Biol. 10.3389/fcell.2023.1228679 37457297 10.3389/fcell.2023.1228679 PMC10340527

[72] Plaza-Oliver M, Santander-Ortega MJ, Lozano MV (2021) Current approaches in lipid-based nanocarriers for oral drug delivery. Drug Deliv Transl Res. 10.1007/s13346-021-00908-7 33528830 10.1007/s13346-021-00908-7 PMC7852471

[73] Costa CP, Moreira JN, Lobo JMS, Silva AC (2021) Intranasal delivery of nanostructured lipid carriers, solid lipid nanoparticles and nanoemulsions: A current overview of in vivo studies. Acta Pharm Sin B. 10.1016/j.apsb.2021.02.012 33996407 10.1016/j.apsb.2021.02.012 PMC8105874

[74] Al-Maghrabi PM, Khafagy ES, Ghorab MM, Gad S (2020) Influence of formulation variables on miconazole nitrate–loaded lipid based nanocarrier for topical delivery. Colloids Surf B Biointerfaces 193. 10.1016/j.colsurfb.2020.111046 10.1016/j.colsurfb.2020.111046 32416518

[75] Rajput AP, Butani SB (2019) Resveratrol anchored nanostructured lipid carrier loaded in situ gel via nasal route: Formulation, optimization and in vivo characterization. J Drug Deliv Sci Technol. 10.1016/j.jddst.2019.01.040

[76] Casati M, Boccardi V, Ferri E, Bertagnoli L, Bastiani P, Ciccone S et al (2020) Vitamin E and Alzheimer’s disease: the mediating role of cellular aging. Aging Clin Exp Res. 10.1007/s40520-019-01209-3 31054115 10.1007/s40520-019-01209-3

[77] Cunha S, Costa CP, Loureiro JA, Alves J, Peixoto AF, Forbes B et al (2020) Double optimization of rivastigmine-loaded nanostructured lipid carriers (NLC) for nose-to-brain delivery using the quality by design (QbD) approach: formulation variables and instrumental parameters. Pharmaceutics 12. 10.3390/pharmaceutics12070599 10.3390/pharmaceutics12070599 PMC7407548 32605177

[78] Wavikar P, Pai R, Vavia P (2017) Nose to brain delivery of Rivastigmine by in situ gelling cationic nanostructured lipid carriers: enhanced brain distribution and pharmacodynamics. J Pharm Sci. 10.1016/j.xphs.2017.08.024 28923321 10.1016/j.xphs.2017.08.024

[79] Sood S, Jain K, Gowthamarajan K (2013) P1–382: Curcumin-donepezil–loaded nanostructured lipid carriers for intranasal delivery in an alzheimer’s disease model. Alzheimer’s Dement. 10.1016/j.jalz.2013.05.609. 9

[80] Gartziandia O, Herran E, Pedraz JL, Carro E, Igartua M, Hernandez RM (2015) Chitosan coated nanostructured lipid carriers for brain delivery of proteins by intranasal administration. Colloids Surf B Biointerfaces. 10.1016/j.colsurfb.2015.06.054 26209963 10.1016/j.colsurfb.2015.06.054

[81] Sadegh Malvajerd S, Izadi Z, Azadi A, Kurd M, Derakhshankhah H, Sharifzadeh M et al (2019) Neuroprotective potential of curcumin-loaded nanostructured lipid carrier in an animal model of alzheimer’s disease: behavioral and biochemical evidence. J Alzheimer’s Dis 69. 10.3233/JAD-190083 10.3233/JAD-190083 31156160

[82] Ullah SNMN, Afzal O, Altamimi ASA, Ather H, Sultana S, Almalki WH et al (2023) Nanomedicine in the management of alzheimer’s disease: State-of-the-art. Biomedicines 11. 10.3390/biomedicines11061752 10.3390/biomedicines11061752 PMC10296528 37371847

[83] Nirale P, Paul A, Yadav KS (2020) Nanoemulsions for targeting the neurodegenerative diseases: Alzheimer’s, parkinson’s and prion’s. Life Sci. 10.1016/j.lfs.2020.117394 32017870 10.1016/j.lfs.2020.117394

[84] Suhail N, Alzahrani AK, Basha WJ, Kizilbash N, Zaidi A, Ambreen J et al (2021) Microemulsions: unique properties, Pharmacological applications, and targeted drug delivery. Front Nanotechnol 3. 10.3389/fnano.2021.754889

[85] Patil RP, Pawara DD, Gudewar CS, Tekade AR (2019) Nanostructured cubosomes in an in situ nasal gel system: an alternative approach for the controlled delivery of donepezil HCl to brain. J Liposome Res. 10.1080/08982104.2018.1552703 30501444 10.1080/08982104.2018.1552703

[86] Kell DB, Heyden EL, Pretorius E (2020) The biology of lactoferrin, an iron-binding protein that can help defend against viruses and bacteria. Front Immunol. 10.3389/fimmu.2020.01221 32574271 10.3389/fimmu.2020.01221 PMC7271924

[87] Kaur A, Nigam K, Srivastava S, Tyagi A, Dang S (2020) Memantine nanoemulsion: a new approach to treat alzheimer’s disease. J Microencapsul 37. 10.1080/02652048.2020.1756971 10.1080/02652048.2020.1756971 32293915

[88] Kaur A, Nigam K, Bhatnagar I, Sukhpal H, Awasthy S, Shankar S et al (2020) Treatment of alzheimer’s diseases using donepezil nanoemulsion: an intranasal approach. Drug Deliv Transl Res 10. 10.1007/s13346-020-00754-z 10.1007/s13346-020-00754-z 32297166

[89] DeRidder L, Sharma A, Liaw K, Sharma R, John J, Kannan S et al (2021) Dendrimer–tesaglitazar conjugate induces a phenotype shift of microglia and enhances β-amyloid phagocytosis. Nanoscale 13. 10.1039/D0NR05958G 10.1039/d0nr05958g 33479718

[90] Jiang Y, Liu C, Zhai W, Zhuang N, Han T, Ding Z (2019) The optimization design of lactoferrin loaded HupA nanoemulsion for targeted drug transport via intranasal route. Int J Nanomed. 10.2147/IJN.S214657 10.2147/IJN.S214657 PMC6885571 31819426

[91] Doshi M (2011) Dendrimer and its application. Int J Pharm Sci Rev Res 7(2):104–111

[92] Ockwig NW, Nenoff TM, Separation H, Rev C, Chem E, Laurent S et al (2010) Additions Corr 110. 10.1021/cr0501792

[93] Winarti L (2015) Sistem Penghantaran Obat tertarget, macam, jenis-jenis sistem Penghantaran, Dan Aplikasinya. STOMATOGNATIC-Jurnal Kedokt Gigi. 10.52643/jbik.v13i3.2678

[94] De R, Mahata MK, Kim K (2022) Structure-based varieties of polymeric nanocarriers and influences of their physicochemical properties on drug delivery profiles. Adv Sci 9. 10.1002/advs.202105373 10.1002/advs.202105373 PMC8981462 35112798

[95] Igartúa DE, Martinez CS, del Alonso V, Prieto S (2020) Combined therapy for Alzheimer’s disease: tacrine and PAMAM dendrimers co-administration reduces the side effects of the drug without modifying its activity. AAPS PharmSciTech. 10.1208/s12249-020-01652-w 32215751 10.1208/s12249-020-01652-w

[96] Gothwal A, Kumar H, Nakhate KT, Ajazuddin, Dutta A, Borah A et al (2019) Lactoferrin coupled lower generation PAMAM dendrimers for brain targeted delivery of memantine in aluminum-chloride-induced alzheimer’s disease in mice. Bioconjug Chem. 10.1021/acs.bioconjchem.9b00505 31553175 10.1021/acs.bioconjchem.9b00505

[97] Jain KK (2009) Application of nanobiotechnology in cancer therapeutics. Pharm Perspect Cancer Ther. ;245–268

[98] Cho K, Wang XU, Nie S, Chen Z, Shin DM (2008) Therapeutic nanoparticles for drug delivery in cancer. Clin Cancer Res. 10.1158/1078-0432.CCR-07-1441 18316549 10.1158/1078-0432.CCR-07-1441

[99] Heinz H, Pramanik C, Heinz O, Ding Y, Mishra RK, Marchon D et al (2017) Nanoparticle decoration with surfactants: molecular interactions, assembly, and applications. Surf Sci Rep 72. 10.1016/j.surfrep.2017.02.001

[100] Vasile C (2018) Polymeric nanomaterials in nanotherapeutics. Elsevier

[101] Wang P, Zheng X, Guo Q, Yang P, Pang X, Qian K et al (2018) Systemic delivery of BACE1 SiRNA through neuron-targeted nanocomplexes for treatment of alzheimer’s disease. J Control Release. 10.1016/j.jconrel.2018.04.034 29679667 10.1016/j.jconrel.2018.04.034

[102] Huo X, Zhang Y, Jin X, Li Y, Zhang L (2019) A novel synthesis of selenium nanoparticles encapsulated PLGA nanospheres with Curcumin molecules for the Inhibition of amyloid β aggregation in alzheimer’s disease. J Photochem Photobiol B Biol 190. 10.1016/j.jphotobiol.2018.11.008 10.1016/j.jphotobiol.2018.11.008 30504054

[103] Yusuf M, Khan M, Alrobaian MM, Alghamdi SA, Warsi MH, Sultana S et al (2021) Brain targeted Polysorbate-80 coated PLGA thymoquinone nanoparticles for the treatment of alzheimer’s disease, with biomechanistic insights. J Drug Deliv Sci Technol. 10.1016/j.jddst.2020.102214

[104] Naki T, Matshe WMR, Balogun MO, Sinha Ray S, Egieyeh SA, Aderibigbe BA (2023) Polymer drug conjugates containing memantine, Tacrine and cinnamic acid: promising nanotherapeutics for the treatment of alzheimer’s disease. J Microencapsul. 10.1080/02652048.2023.2167011 36622880 10.1080/02652048.2023.2167011

[105] Jeon SG, Cha MY, Kim Jil, Hwang TW, Kim KA, Kim TH et al (2019) Vitamin D-binding protein-loaded PLGA nanoparticles suppress alzheimer’s disease-related pathology in 5XFAD mice. Nanomed Nanatechnol Biol Med 17. 10.1016/j.nano.2019.02.004 10.1016/j.nano.2019.02.004 30794963

[106] Nanaki SG, Spyrou K, Bekiari C, Veneti P, Baroud TN, Karouta N et al (2020) Hierarchical porous carbon—plla and Plga hybrid nanoparticles for intranasal delivery of galantamine for alzheimer’s disease therapy. Pharmaceutics. 10.3390/pharmaceutics12030227 32143505 10.3390/pharmaceutics12030227 PMC7150929

[107] Cano A, Ettcheto M, Chang JH, Barroso E, Espina M, Kühne BA et al (2019) Dual-drug loaded nanoparticles of Epigallocatechin-3-gallate (EGCG)/Ascorbic acid enhance therapeutic efficacy of EGCG in a APPswe/PS1dE9 alzheimer’s disease mice model. J Control Release. 10.1016/j.jconrel.2019.03.010 30876953 10.1016/j.jconrel.2019.03.010 PMC6510952

[108] Singh SK, Mishra DN (2019) Nose to brain delivery of galantamine loaded nanoparticles: in-vivo pharmacodynamic and biochemical study in mice. Curr Drug Deliv. 10.2174/1567201815666181004094707 30289074 10.2174/1567201815666181004094707

[109] Mahl CRA, Taketa TB, Rocha-Neto JBM, Almeida WP, Beppu MM (2020) Copper ion uptake by chitosan in the presence of amyloid-β and histidine. Appl Biochem Biotechnol. 10.1007/s12010-019-03120-z 31630339 10.1007/s12010-019-03120-z

[110] Liu Z, Liu Q, Zhang B, Liu Q, Fang L, Gou S (2021) Blood–brain barrier permeable and no-releasing multifunctional nanoparticles for Alzheimer’s disease treatment: targeting NO/cGMP/CREB signaling pathways. J Med Chem. 10.1021/acs.jmedchem.1c01240 34517696 10.1021/acs.jmedchem.1c01240

[111] Sintov AC, Velasco-Aguirre C, Gallardo-Toledo E, Araya E, Kogan MJ (2016) Metal nanoparticles as targeted carriers circumventing the blood–brain barrier. Int Rev Neurobiol. 10.1016/bs.irn.2016.06.007 27678178 10.1016/bs.irn.2016.06.007

[112] Alaqad K, Saleh TA (2016) Gold and silver nanoparticles: synthesis methods, characterization routes and applications towards drugs. J Environ Anal Toxicol. 10.4172/2161-0525.1000384

[113] Lockman PR, Koziara JM, Mumper RJ, Allen DD (2004) Nanoparticle surface charges alter blood–brain barrier integrity and permeability. J Drug Target. 10.1080/10611860400015936 15621689 10.1080/10611860400015936

[114] Hou K, Zhao J, Wang H, Li B, Li K, Shi X et al (2020) Chiral gold nanoparticles enantioselectively rescue memory deficits in a mouse model of Alzheimer’s disease. Nat Commun. 10.1038/s41467-020-18525-2 32963242 10.1038/s41467-020-18525-2 PMC7509831

[115] Dos Santos Tramontin N, da Silva S, Arruda R, Ugioni KS, Canteiro PB, de Bem Silveira G et al (2020) Gold nanoparticles treatment reverses brain damage in alzheimer’s disease model. Mol Neurobiol. 10.1007/s12035-019-01780-w 31612296 10.1007/s12035-019-01780-w

[116] Sanati M, Khodagholi F, Aminyavari S, Ghasemi F, Gholami M, Kebriaeezadeh A et al (2019) Impact of gold nanoparticles on amyloid β-induced alzheimer’s disease in a rat animal model: involvement of STIM proteins. ACS Chem Neurosci. 10.1021/acschemneuro.8b00622 30933476 10.1021/acschemneuro.8b00622

[117] Li Y, Lim E, Fields T, Wu H, Xu Y, Wang YA et al (2019) Improving sensitivity and specificity of amyloid-β peptides and tau protein detection with antibiofouling magnetic nanoparticles for liquid biopsy of Alzheimer’s disease. ACS Biomater Sci Eng. 10.1021/acsbiomaterials.9b00086 33405741 10.1021/acsbiomaterials.9b00086 PMC8720568

[118] Paliwal R, Paliwal SR, Kenwat R, Kurmi B, Das, Sahu MK (2020) Solid lipid nanoparticles: A review on recent perspectives and patents. Expert Opin Ther Pat. 10.1080/13543776.2020.1720649 32003260 10.1080/13543776.2020.1720649

[119] Akel H, Ismail R, Csoka I (2020) Progress and perspectives of brain-targeting lipid-based nanosystems via the nasal route in Alzheimer’s disease. Eur J Pharm Biopharm. 10.1016/j.ejpb.2019.12.014 31926222 10.1016/j.ejpb.2019.12.014

[120] Regina A, Demeule M, Lavallée CCI, Poirier J, Gabathuler R et al (2008) Antitumour activity of ANG1005, a conjugate between Paclitaxel and the new brain delivery vector Angiopep-2. Br J Pharmacol. 10.1038/bjp.2008.260 18574456 10.1038/bjp.2008.260 PMC2538693

[121] Samudre S, Tekade A, Thorve K, Jamodkar A, Parashar G, Chaudhari N (2015) Xanthan gum coated mucoadhesive liposomes for efficient nose to brain delivery of curcumin. Drug Deliv Lett 5(3):201–207

[122] Li W, Zhou Y, Zhao N, Hao B, Wang X, Kong P (2012) Pharmacokinetic behavior and efficiency of acetylcholinesterase inhibition in rat brain after intranasal administration of Galanthamine hydrobromide loaded flexible liposomes. Environ Toxicol Pharmacol. 10.1016/j.etap.2012.04.012 22613079 10.1016/j.etap.2012.04.012

[123] Fernandes M, Lopes I, Magalhães L, Sárria MP, Machado R, Sousa JC et al (2021) Novel concept of exosome-like liposomes for the treatment of alzheimer’s disease. J Control Release. 10.1016/j.jconrel.2021.06.018 34126168 10.1016/j.jconrel.2021.06.018

[124] Hernandez C, Shukla S (2022) Liposome based drug delivery as a potential treatment option for Alzheimer’s disease. Neural Regen Res. 10.4103/1673-5374.327328 34782553 10.4103/1673-5374.327328 PMC8643057

[125] Dos Santos Rodrigues B, Kanekiyo T, Singh J (2019) ApoE-2 brain-targeted gene therapy through transferrin and penetratin tagged liposomal nanoparticles. Pharm Res. 10.1007/s11095-019-2691-7 31529284 10.1007/s11095-019-2691-7 PMC10150442

[126] Andrade S, Pereira MC, Loureiro JA (2023) Caffeic acid loaded into engineered lipid nanoparticles for Alzheimer’s disease therapy. Colloids Surf B Biointerfaces. 10.1016/j.colsurfb.2023.113270 36996633 10.1016/j.colsurfb.2023.113270

[127] Saffari PM, Alijanpour S, Takzaree N, Sahebgharani M, Etemad-Moghadam S, Noorbakhsh F et al (2020) Metformin loaded phosphatidylserine nanoliposomes improve memory deficit and reduce neuroinflammation in streptozotocin-induced alzheimer’s disease model. Life Sci. 10.1016/j.lfs.2020.117861 32473247 10.1016/j.lfs.2020.117861

[128] Kong L, Li X, tao, Ni Y nan, Xiao H, he, Yao Y, jia, Wang Y et al (2020) yuan,. Transferrin-modified osthole PEGylated liposomes travel the blood-brain barrier and mitigate Alzheimer’s disease-related pathology in APP/PS-1 mice. Int J Nanomedicine. ;10.2147/IJN.S239608 10.2147/IJN.S239608 PMC7186891 32425521

[129] Kocsis I, Sanna E, Hunter CA (2021) Liposome enhanced detection of amyloid protein aggregates. Org Lett. 10.1021/acs.orglett.0c03597 33467854 10.1021/acs.orglett.0c03597

[130] Yang P, Sheng D, Guo Q, Wang P, Xu S, Qian K et al (2020) Neuronal mitochondria-targeted micelles relieving oxidative stress for delayed progression of alzheimer’s disease. Biomaterials 238. 10.1016/j.biomaterials.2020.119844 10.1016/j.biomaterials.2020.119844 32062148

[131] Agwa MM, Abdelmonsif DA, Khattab SN, Sabra S (2020) Self-assembled lactoferrin-conjugated linoleic acid micelles as an orally active targeted nanoplatform for Alzheimer’s disease. Int J Biol Macromol. 10.1016/j.ijbiomac.2020.06.058 32531361 10.1016/j.ijbiomac.2020.06.058

[132] Reading CL, Ahlem CN, Murphy MF (2021) NM101 phase III study of NE3107 in alzheimer’s disease: Rationale, design and therapeutic modulation of neuroinflammation and insulin resistance. Neurodegener Dis Manag 11. 10.2217/nmt-2021-0022 10.2217/nmt-2021-0022 34251287

[133] Webster L, Groskreutz D, Grinbergs-Saull A, Howard R, O’Brien JT, Mountain G et al (2017) Core outcome measures for interventions to prevent or slow the progress of dementia for people living with mild to moderate dementia: systematic review and consensus recommendations. PLoS ONE. 10.1371/journal.pone.0179521 28662127 10.1371/journal.pone.0179521 PMC5491018

[134] Aisen PS, Cummings J, Jack CR, Morris JC, Sperling R, Frölich L et al (2017) On the path to 2025: Understanding the alzheimer’s disease continuum. Alzheimers Res Ther. 10.1186/s13195-017-0283-5 28793924 10.1186/s13195-017-0283-5 PMC5549378

[135] Kumar N, Kumar R (2013) Nanotechnology and nanomaterials in the treatment of Life-threatening diseases. 10.3390/cells12232669. William Andrew

[136] Vilella A, Belletti D, Sauer AK, Hagmeyer S, Sarowar T, Masoni M, Stasiak N, Mulvihill JJE, Ruozi B, Forni F, Vandelli MA, Tosi G, Zoli M, Grabrucker AM (2018) Reduced plaque size and inflammation in the APP23 mouse model for alzheimer’s disease after chronic application of polymeric nanoparticles for CNS targeted zinc delivery. J Trace Elem Med Biol 49. 10.1016/j.jtemb.2017.12.006 10.1016/j.jtemb.2017.12.006 29325805

[137] Gao F, Zhao J, Liu P, Ji D, Zhang L, Zhang M, Li Y, Xiao Y (2020) Preparation and in vitro evaluation of multi-target-directed selenium-chondroitin sulfate nanoparticles in protecting against the alzheimer’s disease. Int J Biol Macromol 142. 10.1016/j.ijbiomac.2019.09.098 10.1016/j.ijbiomac.2019.09.098 31593732

[138] El-Hawwary SS, Abd Almaksoud HM, Saber FR, Elimam H, Sayed AM, El Raey MA, Abdelmohsen UR (2021) Green-synthesized zinc oxide nanoparticles, anti-Alzheimer potential and the metabolic profiling of Sabal Blackburniana grown in Egypt supported by molecular modelling. RSC Adv. 10.1039/d1ra01725j 35480186 10.1039/d1ra01725j PMC9033216

[139] Cáceres C, Heusser B, Garnham A, Moczko E The Major Hypotheses of Alzheimer’s Disease: Related Nanotechology-Based Approaches for Its Diagnosis and Treatment.Cells.2023;12 10.3390/cells12232669 PMC10705786 38067098

